# Adverse clinical outcomes and immunosuppressive microenvironment of RHO-GTPase activation pattern in hepatocellular carcinoma

**DOI:** 10.1186/s12967-024-04926-0

**Published:** 2024-01-31

**Authors:** Qi Yang, Zewei Zhuo, Xinqi Qiu, Ruibang Luo, Kehang Guo, Huihuan Wu, Rui Jiang, Jingwei Li, Qizhou Lian, Pengfei Chen, Weihong Sha, Hao Chen

**Affiliations:** 1 Department of Gastroenterology, Guangdong Provincial People’s Hospital (Guangdong Academy of Medical Sciences), Southern Medical University, Guangzhou, 510080 Guangdong China; 2 The Second School of Clinical Medicine, Southern Medical University, Guangzhou, China; 3 School of Medicine, South China University of Technology, Guangzhou, 510006 Guangdong China; 4 Cancer Prevention Center, State Key Laboratory of Oncology in South China, Guangdong Provincial Clinical Research Center for Cancer, Sun Yat-Sen University Cancer Center, Guangzhou, 510060 People’s Republic of China; 5 Department of Computer Science, The University of Hong Kong, Hong Kong, 999077 SAR China; 6 Department of Critical Care Medicine, The Fifth Affiliated Hospital of Zhengzhou University, Zhengzhou, 450000 Henan China; 7 Faculty of Synthetic Biology, Shenzhen Institute of Advanced Technology, Chinese Academy of Sciences, Shenzhen, 518118 Guangdong China; 8 Cord Blood Bank, Guangzhou Institute of Eugenics and Perinatology, Guangzhou Women and Children’s Medical Center, Guangzhou Medical University, Guangzhou, 511436 Guangdong China; 9 State Key Laboratory of Pharmaceutical Biotechnology, The University of Hong Kong, Hong Kong, 999077 SAR China; 10 Department of Laboratory Medicine, State Key Laboratory of Oncology in South China, Collaborative Innovation Center for Cancer Medicine, Sun Yat-Sen University Cancer Center, Guangzhou, 510060 People’s Republic of China

**Keywords:** Rho GTPases, Hepatocellular carcinoma, Pan-cancer, Immune infiltration, Single-cell transcriptome

## Abstract

**Background:**

Emerging evidence suggests that Rho GTPases play a crucial role in tumorigenesis and metastasis, but their involvement in the tumor microenvironment (TME) and prognosis of hepatocellular carcinoma (HCC) is not well understood.

**Methods:**

We aim to develop a tumor prognosis prediction system called the Rho GTPases-related gene score (RGPRG score) using Rho GTPase signaling genes and further bioinformatic analyses.

**Results:**

Our work found that HCC patients with a high RGPRG score had significantly worse survival and increased immunosuppressive cell fractions compared to those with a low RGPRG score. Single-cell cohort analysis revealed an immune-active TME in patients with a low RGPRG score, with strengthened communication from T/NK cells to other cells through MIF signaling networks. Targeting these alterations in TME, the patients with high RGPRG score have worse immunotherapeutic outcomes and decreased survival time in the immunotherapy cohort. Moreover, the RGPRG score was found to be correlated with survival in 27 other cancers. In vitro experiments confirmed that knockdown of the key Rho GTPase-signaling biomarker SFN significantly inhibited HCC cell proliferation, invasion, and migration.

**Conclusions:**

This study provides new insight into the TME features and clinical use of Rho GTPase gene pattern at the bulk-seq and single-cell level, which may contribute to guiding personalized treatment and improving clinical outcome in HCC.

**Supplementary Information:**

The online version contains supplementary material available at 10.1186/s12967-024-04926-0.

## Introduction

Primary liver cancer (PLC) is the sixth frequently diagnosed cancer and the third leading cause of cancer-related mortality, seriously threatening human life and health [1]. As the most predominant type of PLC, hepatocellular carcinoma (HCC) is known to be a highly aggressive and heterogeneous tumor with high recurrence rate and shorter 5-year survival rate of less than 20% [2]. One-third of HCC cases diagnosed at an advanced stage due to the absence of screening or follow-up [3], and thus local and systemic metastasis are the primary causes leading to a failure treatment and dismal prognosis of HCC [4]. Although the clinical benefits of immune checkpoint inhibitor (ICI) are emerging for patients with advanced HCC, the disappointing results of some phase 2 and 3 trials for HCC immunotherapy represent continued challenges [5, 6]. In addition, it has been reported that tumor response to ICI associates with prognosis, which underscores the need for accurate biomarkers of the risk stratification and treatment response in the clinical management of HCC.

Rho GTPases are crucial signal transducers regulating multiple biological processes involved in cell polarity, adhesion, and migration, contributing to cancer cell invasiveness and metastasis [7]. The transformation process of primary cancers to metastatic cancers has high relatedness with the activation of Rho GTPases pathway, and this could be attributed to cooperative interaction between Rho GTPases pathway and proto-oncogene (K-Ras and H-Ras) [8, 9]. For instance, activated Rho GTPase members facilitate bladder cancer [10], breast cancer [11], and colon cancer [12] metastasis. Some Rho GTPases pathway-relate genes such as RHOA, RAC1, PAK1, and ARHGEF38 have also been indicated to play critical roles in metastatic cancer at an advanced stage [13–16], which provides promising for cancer-specific treatments. Notably, emerging study has linked Rho GTPases to tumor immunity, such as a blockade of Rho-kinase (ROCK), which could induce anti-tumor immunity by increasing cancer cell phagocytosis and dendritic cell (DC)-mediated T cell priming [17]. In immunosuppressed environments, abnormal alterations of the Rho GTPases pathway have been reported to exert oncogenic role in lymphoma [18], whereas the immune infiltration and clinical significance of Rho GTPases pathway-related genes in HCC have not been fully elucidated.

**Fig. 1:**
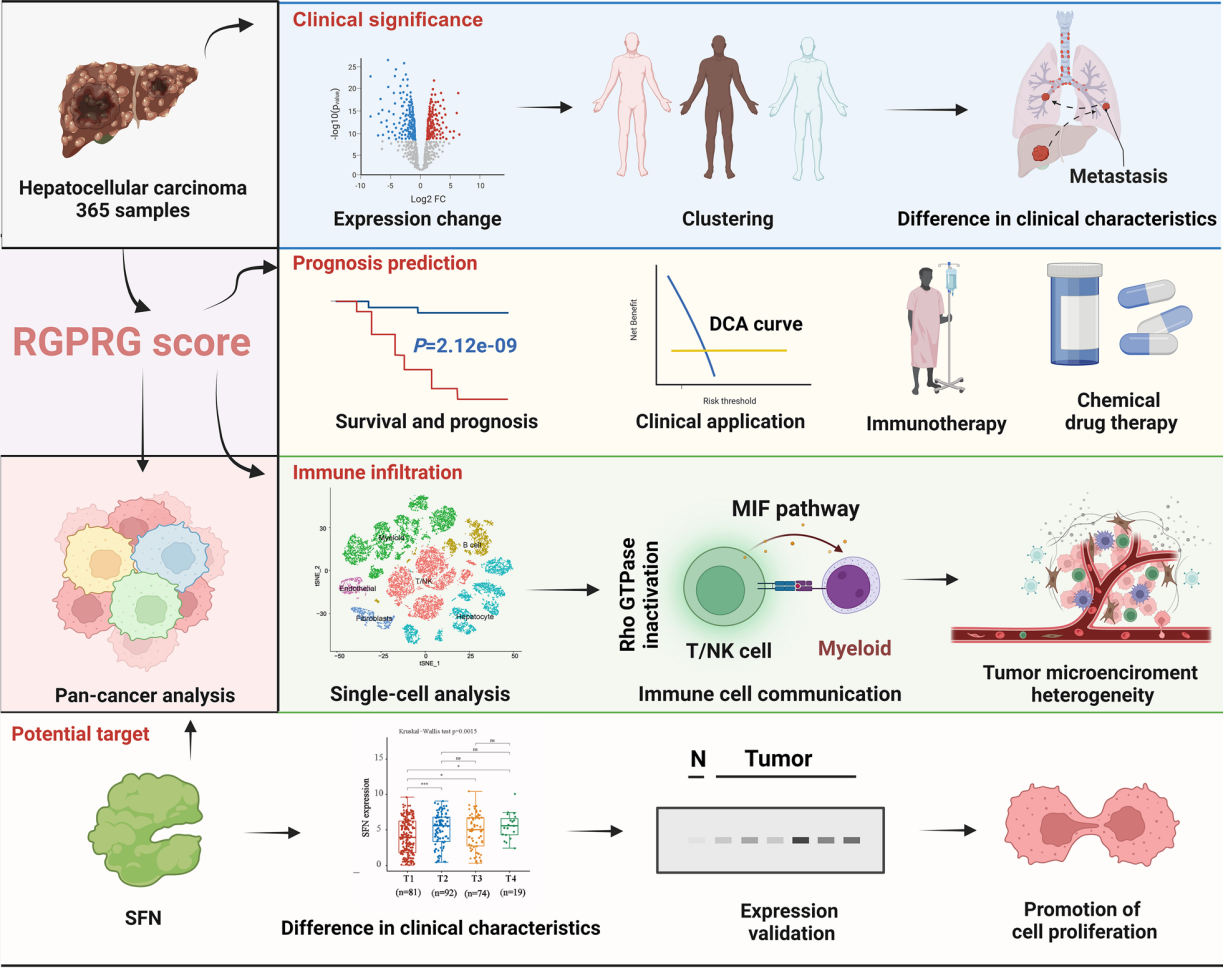
Schematic design of Rho GTPases gene expression pattern to assess the connection between Rho GTPases and immune infiltration, immunotherapy response, chemotherapeutic agent selection, and prognosis in HCC

Considering the significance of the role of Rho GTPases in metastatic cancers and tumor immunity, we put forward the hypothesis that Rho GTPases gene expression pattern may affect the clinical outcome and tumor immune infiltration in HCC patients. In this study, we attempted to conduct a multi-level bioinformatic analysis on Rho GTPases signaling-related genes in HCC patients from 3 independent public datasets, which aim to provide a connection between Rho GTPases and immune infiltration, immunotherapy response, chemotherapeutic agent selection, and prognosis in HCC (see Fig. 1). This study may contribute to optimizing precision treatment and improving clinical outcome of HCC patients.

## Material and methods

### Data collection and processing

RNA-sequencing data and corresponding clinical information of HCC patients were obtained from three different cohorts: The Cancer Genome Atlas (TCGA, https://portal.gdc.cancer.gov/) (TCGA-LIHC), International Cancer Genome Consortium (ICGC, https://dcc.icgc.org/) (ICGC-LIRI-JP), and Gene Expression Omnibus (GEO, http://www.ncbi.nlm.nih.gov/geo) (GSE76427). The patient characteristics were displayed in Table 1. Immunotherapeutic response data (anti-PD-L1 therapy) were obtained from IMvigor210 database. A single-cell cohort of HCC samples was obtained from GEO database (GSE149614). 428 Rho GTPase-signaling genes were collected from molecular signatures database (MSigDB) on Feb 21st, 2022, listed in Additional file 2: Table S1. The study design was in line with the REMARK criteria [19].

**Table 1:** Clinicopathologic characteristics HCC patients

	TCGA-LIHC	ICGC-LIRI-JP	GSE76427
No. of patients	365	243	115
Age (%)			
< 45	42 (11.5%)	5 (2.1%)	7 (6.08%)
≥ 45	323 (88.5%)	238 (97.9%)	108 (93.92%)
Gender (%)			
Female	119 (32.6%)	61 (25.1%)	22 (19.13%)
Male	246 (67.4%)	182 (74.9%)	93 (80.87%)
Grade (%)			
Grade 1	55 (15.1%)	30 (12.3%)	50 (43.5%)
Grade 2	175 (47.9%)	152 (62.3%)	
Grade 3	118 (32.3%)	20 (8.2%)	62 (53.9%)
Grade 4	12 (3.3%)	1 (0.4%)	
Unknown	5 (1.4%)	40 (16.8%)	3 (2.6%)
Tumor stage (%)			
I	170 (46.6%)	36 (14.8%)	55 (47.83%)
II	84 (23.0%)	110 (45.3%)	35 (30.43%)
III	83 (22.7%)	76 (31.3%)	21 (18.26%)
IV	4 (1.09%)	21 (8.6%)	3 (2.61%)
Unknown	24 (6.6%)		1 (0.87%)
T stage (%)			
T1	180 (49.3%)	NR	NR
T2	91 (24.9%)	NR	NR
T3	78 (21.4%)	NR	NR
T4	13 (3.6%)	NR	NR
Tx	1 (0.3%)	NR	NR
Unknown	2 (0.5%)	NR	NR
N stage (%)			
N0	248 (67.9%)	NR	NR
N1	4 (1.09%)	NR	NR
Nx	112 (30.7%)	NR	NR
Unknown	1 (0.3%)	NR	NR
M stage (%)			
M0	263 (72.1%)	NR	NR
M1	3 (0.8%)	NR	NR
Mx	99 (27.1%)	NR	NR
Survival status			
OS days (median)	556	780	423.4
Censored (%)	126 (34.5%)	44 (18.1%)	23 (20%)

*NR* not reported

### Screening candidate genes

Differentially expressed genes (DEGs) were identified between normal and HCC samples using the “limma” package of R, and “False discovery rate (FDR) < 0.05, and log2|Fold change|> 1” was set as a threshold. Univariate regression analysis was used for the analysis of prognosis-related genes, and P < 0.05 was set as a threshold for significance. The common candidate genes were obtained using the “VeenDiagram” package of R and then were visualized using “pheatmap” package of R. Protein–protein interaction (PPI) networks for candidate genes were analyzed using the STRING database (Version: 12.0, http://string-db.org).

### Consensus clustering analysis

To further discover molecular cancer subtype based on the expression of Rho GTPase-signaling genes, HCC samples were drawn for a repetitive test, and different clusters’ solutions (k = 2 to k = 6) was calculated using the “ConsensusClusterPlus” package of R to find the optimal number of clusters. Clustering heatmaps and gene expression heatmap in subgroups were plotted using “pheatmap” package of R. Kaplan–Meier survival analysis was performed to compare the survival difference between different subgroups.

### Rho GTPase-signaling prognostic scoring system

Least absolute shrinkage and selection operator (LASSO) regression analysis was conducted on the candidate genes for the optimal feature genes selection and model construction by a tenfold cross-validation method. Once the model is selected, use the coefficients or weights assigned to each gene in the model to calculate individual risk score for each HCC patients. We defined such a tumor prognosis prediction system called the Rho GTPases-related gene score (RGPRG score). The RGPRG score could be calculated through the following formula:

$$\mathrm{RGPRG\,score}=\sum_{i=1}^{n}Xi\times Yi,$$

where X represents risk coefficients and Y represents gene expression level. Based on the median RGPRG score, HCC samples could divide into high- and low-RGPRG score groups. The Receiver Operating Characteristic (ROC) curve analysis was applied to compare the predictive accuracy of the prognostic scoring system using the “timeROC” package of R. Two external cohorts (ICGC-LIRI-JP and GSE149614) were used to validate the predictive ability and reliability of the prognostic scoring system.

### Clinical significance

Clinical characteristics (including age, sex, Grade, Stage, T stage, M stage, N stage) and RGPRG score were analyzed to identify prognosis-related factors by univariate and multivariate regression analyses. The correlations between RGPRG score and clinical characteristics were further estimated using the Wilcoxon rank sum test. Decision curve analysis (DCA) was used to evaluate and compare multiple published clinical prediction models [20–23]. None and ALL are two reference lines, and the closer the curves of other models are to these two lines, the less clinical utility they have.

### Biofunction analyses

To further reveal the function of the Rho GTPase-signaling gene signature, DEGs were identified between high- and low-RGPRG score groups of HCC patients from TCGA cohort. Gene Ontology (GO) and Kyoto Encyclopedia of Genes and Genomes (KEGG) datasets (“c5.go.v7.4.symbols.gmt” and c2.cp.kegg.v7.4.symbols.gmt”) were downloaded from Gene Set Enrichment Analysis (GSEA) website and were conducted on DEGs for functional and pathway enrichment using "org.Hs.eg.db", " enrichplot", and "clusterProfiler" packages of R.

### Tumor immune landscape analysis

We obtained the CIBERSORT R source code and the LM22 matrix (22 immune cell gene signature) from the CIBERSORT web portal (http://cibersort.stanford.edu/) as previously described. Based on the HCC RNA-seq expression data from TCGA cohort, we used CIBERSORTR algorithm to calculate 22 tumor immune cell proportions, and histogram and boxplot diagrams were produced to visualize the abundance of immune infiltration. To explore the relationship between RGPRG score and the immune checkpoints, a boxplot of the distribution of the immune-checkpoint gene expression values in the high- and low-RGPRG score group was drawn using “ggplot2” packages of R.

### Mutational landscape analysis

Masked somatic mutation data were downloaded from TCGA online database, and the mutation information was extracted and then grouped based on the RGPRG score. Waterfall plots were drawn to visualize mutational landscapes of HCC patients with different RGPRG score groups using the “maftools” package of R.

### Single-cell RNA-seq data analysis

The “Seurat” package of R was used for single-cell quality control (QC) and cell clustering analysis, and the function “AddModuleScore” of the R package “Seurat” was performed to calculate RGPRG score. Different subclusters and different RGPRG scores for each single cell were then displayed in the t-SNE plot. The intercellular communication networks and differential ligand-receptor pairs were identified using the R package “CellChat”.

### Therapeutic response prediction

Based on tumor mutation burden (TMB), Tumor Immune Dysfunction and Exclusion (TIDE) scores, and PD-L1 protein expression, the potential immunotherapy response prediction performance of HCC was assessed. The prediction of different RGPRG score groups to immunotherapeutic efficacy was evaluated using the clinical cohort IMvigor210. Kaplan–Meier method was used to compare the survival difference between two RGPRG score groups in the immunotherapeutic cohort. The Genomics of Drug Sensitivity in Cancer (GDSC), the largest public pharmacogenomic database, was used to predict drug sensitivity for every HCC sample. The top 10 chemotherapeutic agents optimal for each RGPRG score group were displayed by sorting the p-value.

### Pan-cancer prognosis analyses

Based on the optimal feature genes, we used multivariate cox regression analysis to construct the prognostic model for 35 different cancers including 10,228 patients from TCGA database. The distribution of RGPRG score in pan-cancer patients was displayed in the boxplot. Kaplan–Meier curve was used to compare the prognosis difference in different RGPRG score groups of pan-cancer patients.

### Cell culture

Human liver cell line L02 and six HCC cell lines including Huh7, SK-hep1, Hep3B, SMMC7721, HCCLM3, and HepG2 were purchased from Zhongqiaoxinzhou (Shanghai, China). At 37 °C and 5% CO_2_, all cell lines were cultivated in the Dulbecco's Modified Eagle Medium (DMEM) with 10% fetal bovine serum (FBS) and 1% penicillin/streptomycin (Thermo Fisher Scientific, MA, USA).

### RNA extraction, reverse transcription, and quantitative reverse transcription polymerase chain reaction (qRT-PCR)

Total RNA from cells was extracted by E.Z.N.A. Total RNA Isolation Kit (Omega, GA, USA). The generation of cDNAs from reverse transcription was performed by PrimeScript™ RT-PCR kit (TaKaRa, Otsu, Japan). According to manufacturer instructions of Biorad CFX Connect (Bio-Rad Laboratories, CA, USA), we conducted the qRT-PCR by using SYBR Premix Ex Taq (TaKaRa, Otsu, Japan). The specific operation steps of qRT-PCR were performed as described previously [24]. The primers of SFN and GAPDH are as follows: SFN Forward (5′-ACTTTTCCGTCTTCCACTACGA-3′), Reverse (5′-ACAGTGTCAGGTTGTCTCGC-3′); GAPDH Forward (5′-GGAGCGAGATCCCTCCAAAAT-3′), Reverse (5′-GGCTGTTGTCATACTTCTCATGG-3′).

### Western blot

The proteins of each cell line were lysed with radioimmunoprecipitation assay buffer (RIPA) cell lysis buffer, protease inhibitors, and phosphatase inhibitors, and then the total protein amount was determined by the Bicinchoninic Acid Assay (BCA) method. Protein samples were electrophoresed on 10% SDS-PAGE gels and then transferred to the polyvinylidene fluoride (PVDF) membrane. The membrane was blocked with 5% non-fat milk, and probed with primary antibodies overnight at 4 °C. Membrane was washed three times, followed immediately by incubation with the secondary antibody for 1 h. Detection of the bands was achieved by adding enhanced chemiluminescent (ECL) chromogenic substrate.

### siRNA transfection

SFN siRNA was designed and synthesized by Jijie Biological Technology (Guangzhou, China). According to the manufacturer’s instructions, siRNA and RNAiMAX reagent (Thermo Fisher Scientific, Shanghai, China) were diluted with Opti-MEM medium (Thermo Fisher Scientific, Shanghai, China). Before transfection, cells (1.5 × 10^5^) were seeded into a 6-well plate at 24 h, and then transfection was conducted when reaching 60–70% confluence.

### CCK-8 proliferation assay

Cell Counting Kit-8 (CCK-8) was purchased from Keygen Biotech (Jiangsu, China). Firstly, cells (1 × 10^4^) were seeded into a 96-well plate until reaching 80% confluence. According to the manufacturer’s instructions, 10 μl CCK-8 solution was added into cells followed by 2 h incubation. A microplate spectrophotometer was applied to measure the optical density at 450 nm.

### Cell invasion and migration assays

The cell invasion assay was conducted using a 24-well plate and Transwell chambers. Transfected cells were uniformly seeded in the upper chamber without serum, while the lower chamber of the 24-well plate contained culture medium with 20% FBS as a chemoattractant to induce cell invasion. Crystal violet staining was employed to stain and count cells. For the cell migration assay, cells transfected with targeted siRNA were dispersed into six wells under serum-free conditions. A wound was created using a 100 mL plastic pipette tip. The migration ability was determined by measuring the migration distance after 24 h using the scratch assay method.

### Statistical analysis

All analyses are performed by R software (version 4.1.0). FDR-adjusted p-values were determined for differential expression analyses using the Benjamini–Hochberg method. The difference between two comparisons was evaluated by the Wilcox rank sum test, and multiple comparisons were performed by ANOVA test. The relationship between metric variables was examined by the Spearman correlation. When P value correction for multiple comparisons was required, the Bonferroni method was used.

## Results

### Rho GTPase signaling-related gene transcriptomic landscape and molecule subtype of HCC

**Fig. 2:**
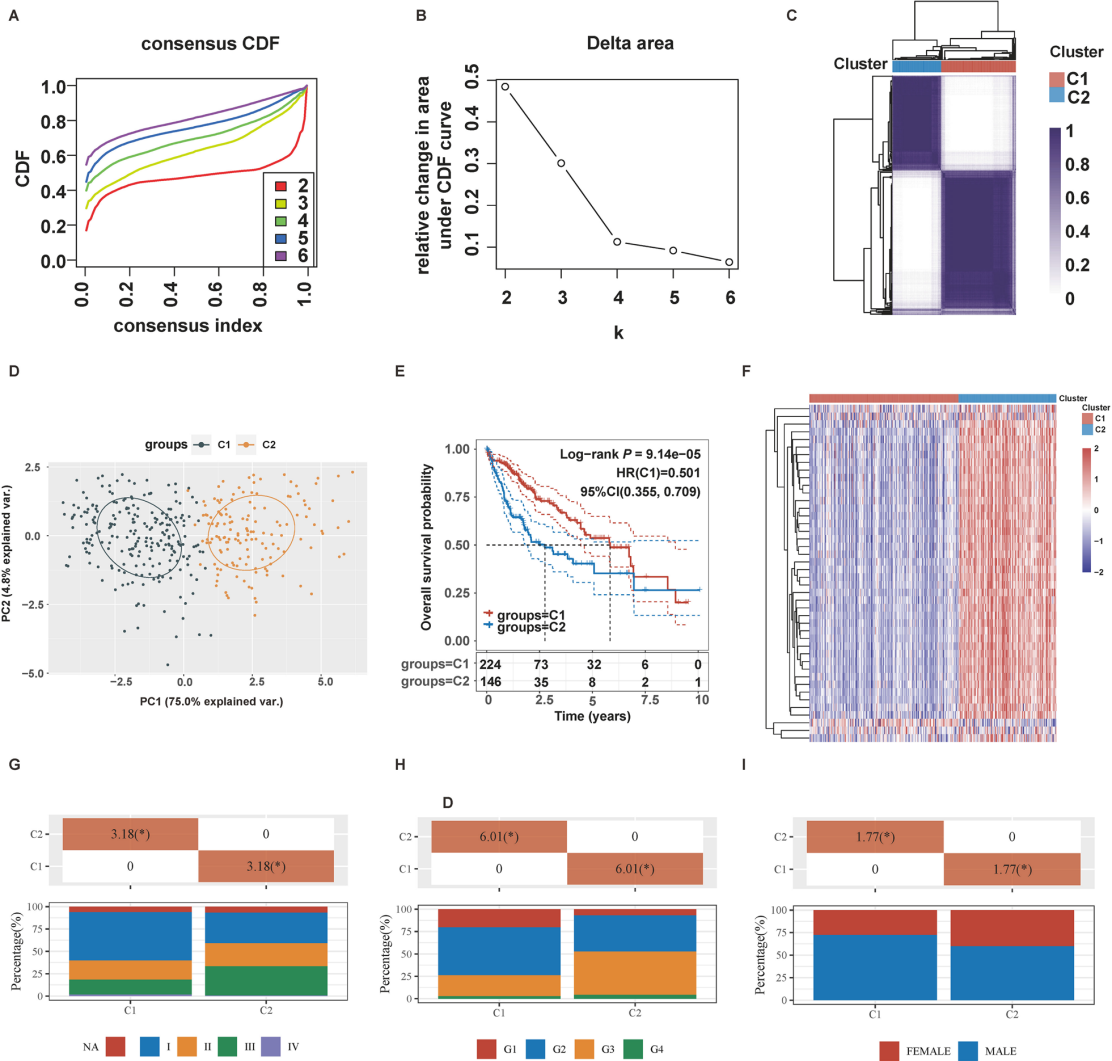
Identifying molecular subtypes of HCC based on 44 candidate Rho GTPase-related genes. **A** Consensus clustering cumulative distribution function (CDF) for k = 2 to 6. **B** When K = 2, the relative change in area under the CDF curve was optimal. **C** consensus clustering identified two clusters. **D** Principal component analysis (PCA) of cluster 1 and cluster 2. **E** Kaplan–Meier (KM) survival analysis of cluster 1 and cluster 2, and cluster 1 had better survival compared with cluster 2. **F** Heatmap of differential expression genes between two clusters. **G** Cluster 1 had a higher proportion of TNM stage I than cluster 2. **H** Cluster 1 had a higher proportion of Grade 1 and 2 than cluster 2. **I** Cluster 1 had a higher proportion of male than cluster 2. *P < 0.05

A total of 365 HCC samples and 50 normal samples with RNA-seq expression data and clinical information were collected from TCGA cohort as a training set while ICGC-LIRI-JP containing 243 HCC patients and GSE76427 containing 115 HCC patients were employed for model validation. These three cohorts themselves demonstrate outcomes that correlate with conventional/classic tumor prognostic factors (TCGA-LIHC, P = 1.1308 × 10^–7^; ICGC-LIRI-JP, P = 5.1141 × 10^–6^; GSE76427, P = 0.0191), allowing us to draw conclusions of prognostic significance (Additional file 1: Fig. S1). 43 candidate Rho GTPase signaling-related genes were obtained by taking the intersection of 67 DEGs (Additional file 2: Table S2) and 180 prognosis-related genes (Additional file 2: Table S3, Additional file 1: Fig. S2A). Compared to the normal samples, 39 Rho GTPase signaling-related genes were up-regulated, and only 4 genes such as CYBB, SYDE2, ARHGEF26, and DEPDC7 were down-regulated in HCC samples (Additional file 1: Fig. S2B). To further identify the hub gene, PPI analysis was conducted on 43 candidate genes, and CENPE, BUB1B, AURKB, CDC20, CENPF, and CDCA8 were the top 6 hub genes ranked by degree (Additional file 1: Fig. S2C). Furthermore, we sought to explore how the imbalanced expression of Rho GTPase signaling-related genes would affect clinical prognosis and the progression of HCC. Consensus Cumulative Distribution Function (CDF) Plot and CDF Delta area suggested that the optimal number of clusters was K = 2 (Fig. 2A, B). HCC patients could be divided into two molecule subtypes including 224 samples in Rho GTPase signaling-related cluster 1 and 146 samples in Rho GTPase signaling-related cluster 2 (Fig. 2C). Principal component analysis (PCA) analysis further confirmed a good separation between cluster 1 and cluster 2 (Fig. 2D). Then, we explore the clinical significance of Rho GTPase signaling-related regulation pattern, and we found that patients of cluster 1 had better survival compared with patients of cluster 2 (*P* = 9.14e−05, Fig. 2E). The transcriptomic landscape of DEGs regulated by two Rho GTPase signaling-related clusters was displayed in the heatmap (Fig. 2F). Moreover, we investigate the correlation between the two clusters and clinical characteristics. Patients of cluster 1 had a higher proportion of tumor, nodes, metastasis-classification (TNM) stage I, Grade 1–2, and a lower proportion of TNM stage III, Grade 3 than cluster 2 (Fig. 2G, H), indicating that Rho GTPase signaling-related genes were strongly associated with the differentiation and progression of advanced HCC. Given the recent debate on whether cancer immunotherapy or chemotherapy efficacy is different between male and female patients [25], we further explore whether the Rho GTPase-identified molecular subtypes signature is different between the male and female patients. Interestingly, we found that the proportion of males in cluster 1 was significantly higher than in cluster 2 (Chi-square value = 1.77, *P* < 0.05, Fig. 2I), suggesting that the immune and clinical significance of Rho GTPase-identified molecular subtypes may differ between males and females.

### Rho GTPase signaling-related gene signature for prediction of HCC prognosis

**Fig. 3:**
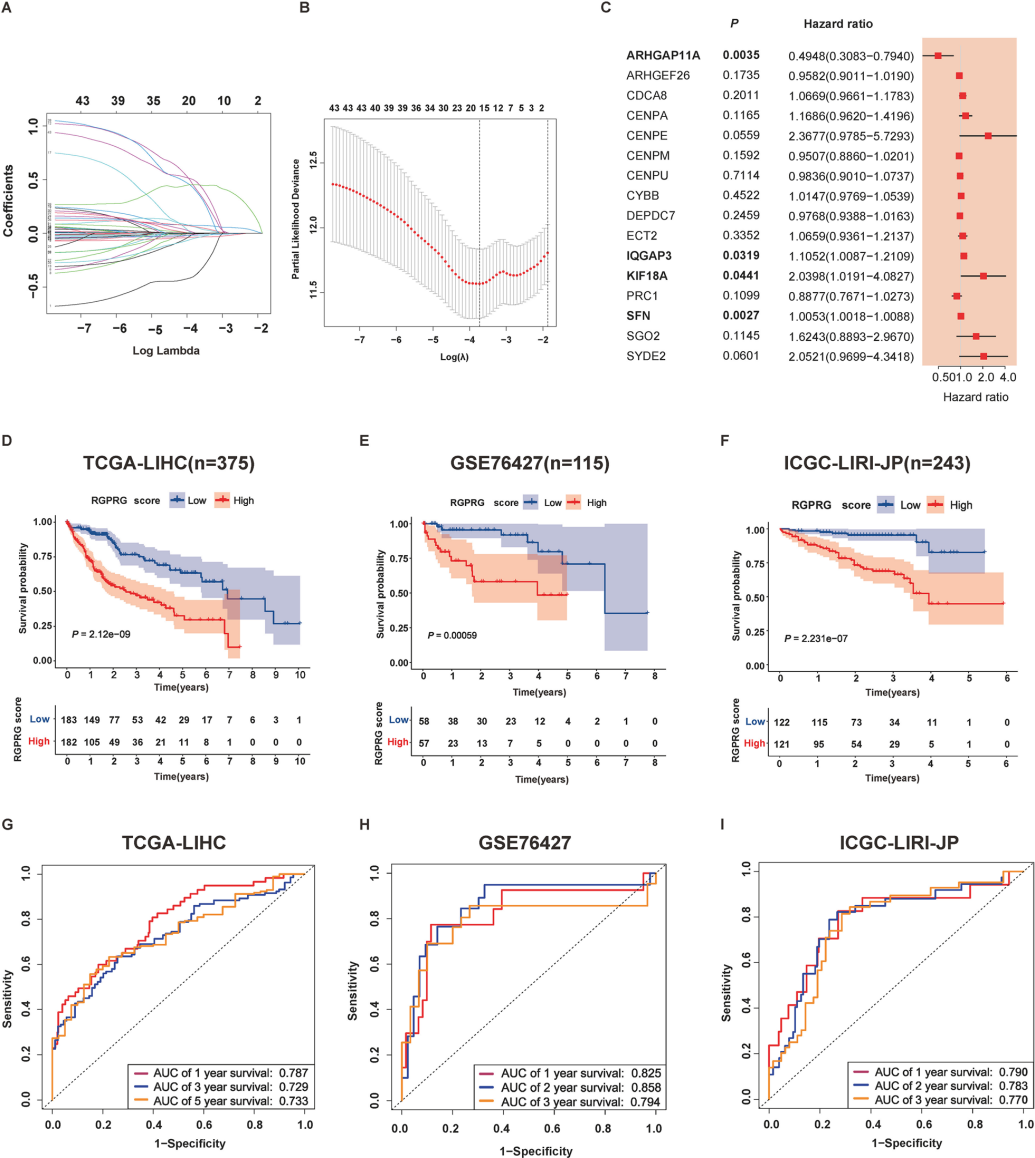
Construction and validation of Rho GTPase-related gene signature in the HCC cohort. **A** The coefficients of selected features in the LSSSO regression model. **B** Partial likelihood deviance of selected features in the LSSSO regression model. **C** Multivariate logistic regression analysis shows that ARHGAP11A, IQGAP3, KIF18A, and SFN are independently associated with prognosis (*P* < 0.05). **D**–**F** K–M curve showing the low-RGPRG score group had better survival than the high-RGPRG score group in TCGA-LIHC, GSE76427, ICGC-LIRI-JP cohorts. **G**–**I** The receiver operation characteristic (ROC) curve of the Rho GTPase-related gene signature to predict 1-, 3-, and 5-year survival in TCGA-LIHC, GSE76427, ICGC-LIRI-JP cohorts

LASSO regression is a widely used method for feature selection in high-dimensional data analysis, which can effectively select important features and avoid overfitting. Based on 43 candidate Rho GTPase signaling-related genes, we used the LASSO algorithm to further narrow down gene numbers and construct an optimal prediction model (Fig. 3A, B). 16 gene including *ARHGAP11A*, *ARHGEF26*, *CDCA8*, *CENPA*, *CENPE*, *CENPM*, *CENPU*, *CYBB*, *DEPDC7*, *ECT2*, *IQGAP3*, *KIF18A*, *PRC1*, *SFN*, *SGO2*, *SYDE2* were eventually selected as signature genes. After multivariate analysis, *ARHGAP11A*, *IQGAP3*, *KIF18A*, and *SFN* were identified as independently prognosis-associated genes*.* As shown in the forest plot, the expression level of *IQGAP3*, *KIF18A*, and *SFN* had a significant correlation with poor survival, while ARHGAP11A played an opposite role (Fig. 3C). We defined such a tumor prognosis prediction system called the Rho GTPases-related gene score (RGPRG score). The RGPRG score was calculated with the following formula:

$$\mathrm{RGPRG\,score}= \left(-0.3803665364*{\text{ARHGAP}}11{\text{A}}\right)+\left(-0.0346771228*{\text{ARHGEF}}26\right)+\left(0.0743931047*{\text{CDCA}}8\right)+\left(0.0550284274*{\text{CENPA}}\right)+\left(0.4046724644*{\text{CENPE}}\right)+\left(-0.0078040620*{\text{CENPM}}\right)+\left(-0.0036923344*{\text{CENPU}}\right)+\left(0.0031554041*\mathrm{ CYBB}\right)+\left(-0.0171528676*{\text{DEPDC}}7\right)+\left(0.0001850116*{\text{ECT}}2\right)+\left(0.0590649975*{\text{IQGAP}}3\right)+\left(0.3867412470*\mathrm{ KIF}18{\text{A}}\right)+\left(-0.0412135681*{\text{PRC}}1\right)+\left(0.0033162272*{\text{SFN}}\right)+\left(0.4529556619*{\text{SGO}}2\right)+\left(0.4793704117*{\text{SYDE}}2\right).$$

According to the median RGPRG score, HCC samples were divided into a high-RGPRG score group (n = 182) and a low-RGPRG score group (n = 183). HCC patients in the high-RGPRG score group had a short survival time and more death compared with patients in the low-RGPRG score group (Additional file 1: Fig. S3A). As for survival analysis, high-RGPRG score patients had significantly worse survival than low-RGPRG score patients (*P* = 2.12e−09, Fig. 3D), showing a good prognostic stratification for HCC patients. ROC analysis demonstrated that area under curve (AUC) values of 1-, 3-, and 5-year survival were 0.787, 0.729, and 0.733, respectively (Fig. 3G), further confirming the predictive performance of Rho GTPase signaling-related gene signature. In the validation cohort of GSE76427 and ICGC-LIRI-JP, HCC patients could be divided into a high-RGPRG score group and a low-RGPRG score group as well based on the median RGPRG score (Additional file 1: Fig S3B). Patients with high RGPRG score had more death (Additional file 1: Fig. S3B), and worse survival compared with the patients with low RGPRG score (*P* = 0.00059, Fig. 3E; *P* = 2.231e−07, Fig. 3F). As for ROC analysis, AUC of 1-, 2-, and 3-year survival in the GSE76427 cohort was 0.825, 0.858, 0.794, respectively (Fig. 3H), while AUC of 1-, 2-, and 3-year survival in ICGC-LIRI-JP cohort was 0.790, 0.783, 0.770 respectively (Fig. 3I). These results indicated that our signature could produce an accurate and robust prediction for HCC prognosis.

### Clinical significance of Rho GTPase signaling-related gene signature

**Fig. 4:**
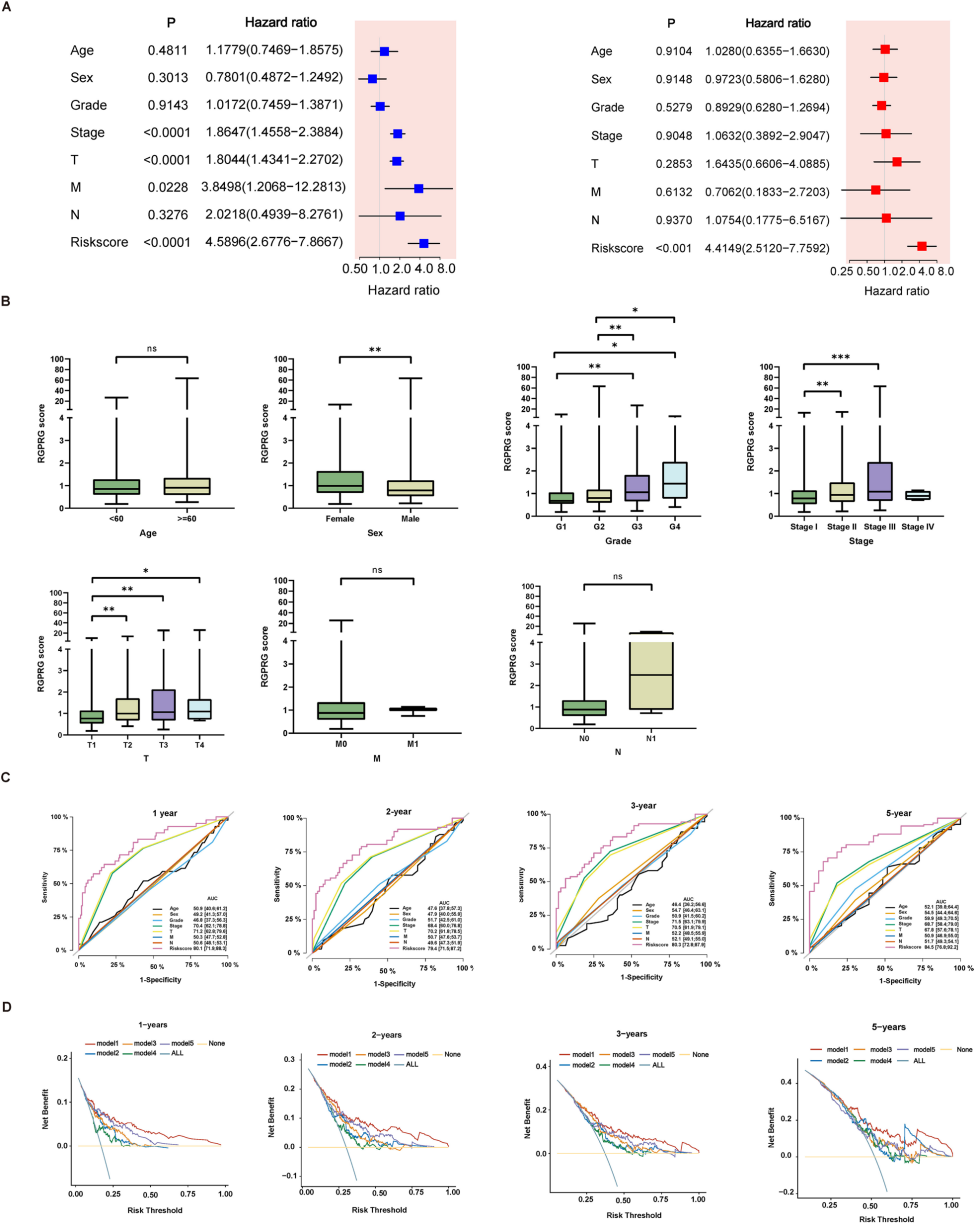
Association of RGPRG score and multiple clinical features. **A** Univariate Cox and Multivariate Cox analyses of the age, sex, grade, stage, T stage, N stage, M stage, and RGPRG score. **B** The relationship between RGPRG score and age, sex, grade, stage, T stage, N stage, M stage. **C** ROC analysis of age, sex, grade, stage, T stage, N stage, M stage, and RGPRG score. **D** The DCA curve for the overall survival rate at 1 year, 2 years, 3 years, and 5 years for the risk model 1 and other risk model. Model 1: Rho GTPase-related risk model; Model 2: pyroptosis-related risk model; Model 3: ferroptosis-related risk model; Model 4: Cuproptosis-related risk model; Model 5: immune-related risk model. *P < 0.05, **P < 0.01, ***P < 0.001

Considering that other clinical characteristics may affect Rho GTPase signaling-related gene signature, we sought to explore the relationship between other clinical characteristics with RGPRG score. After univariate and multivariate analyses, RGPRG score were identified as an independent prognostic factor for HCC patients (all P < 0.001, Fig. 4A). As shown in Fig. 4B, RGPRG score was significantly correlated with sex, grade, stage and T stage. Patients in the high-RGPRG score group accounted for a high proportion of G3, G4, and stage II and III, while patients in the low-RGPRG score group had a high proportion of G1, G2, and stage I (Fig. 4B), suggesting a strong correlation of the RGPRG score and malignancy and advanced tumor of HCC. Compared with other clinical factors, AUC of RGPRG score shows high predictive performance (AUC of 1-year survival = 0.801, AUC of 2-year survival = 0.794, AUC of 3-year survival = 0.803, AUC of 5-year survival = 0.845, Fig. 4C). Furthermore, we used DCA curve to determine whether the model is superior to other strategies (Fig. 4D). Compared with 4 published model, the vertical axis values of total survival rates at 1 year, 2 years, 3 years, and 5 years for the risk model 1 (Rho GTPase signaling-related gene signature) are higher than those for other models in a large threshold range, suggesting that the risk model has better clinical utility.

### High RGPRG score was correlated with cell cycle, primary immunodeficiency, and higher TP53 mutation

To determine the potential regulation pattern of the Rho GTPase signaling-related gene signature, we carried out a GSEA enrichment analysis and found that the two RGPRG score groups showed different biological pathways. In the biological process, patients with low RGPRG score were significantly enriched for the metabolic process including “alpha amino acid catabolic process”, “alpha amino acid metabolic process”, “cellular amino acid catabolic process”, and “cellular lipid catabolic process”, and “complement activation” process (Additional file 1: Fig. S4A), while high RGPRG score was significantly enriched for cell cycle process including “cell division”, “microtubule cytoskeleton organization”, “mitotic nuclear division”, and “organelle fission”, and immune cell migration process such as “leukocyte migration” (Additional file 1: Fig. S4B). In the KEGG enrichment results, the pathway downregulated in HCC patients with low RGPRG score were metabolism pathways including “drug metabolism cytochrome p450”, “fatty acid metabolism”, “glycine serine and threonine metabolism”, and “retinol metabolism”, and “complement and coagulation cascades” pathway (Additional file 1: Fig. S4C), while the pathway upregulated in HCC patients with high RGPRG score were “cell cycle” pathway and immune-related pathways, including “cytokine-cytokine receptor interaction”, “hematopoietic cell lineage”, “neuroactive ligand receptor interaction”, and “primary immunodeficiency” (Additional file 1: Fig. S4D). These results demonstrated that low RGPRG score patients were mainly related to metabolism and complement and coagulation cascades, which may reflect a less aggressive tumor phenotype. In contrast, the upregulated pathways in HCC patients with high RGPRG score were primarily related to cell cycle and immune-related pathways, indicating an active tumor cell proliferation and higher immunosuppression state. Due to the strong correlation between cell cycle and DNA alteration with HCC progression, we further explore the genomic features of RGPRG score in HCC. The somatic alteration occurred in about 84.57% of high RGPRG score patients (Additional file 1: Fig. S5A), whereas this percentage decreased to 73.03% for the patients with low RGPRG score (Additional file 1: Fig. S5B). In terms of gene mutation, TP53 is the most frequently mutated gene in the high RGPRG score patients (37%) compared with low RGPRG score patients (20%), which may account for the reason for poor outcomes in HCC.

### High RGPRG score patients showing higher immunosuppressive tumor microenvironment

**Fig. 5:**
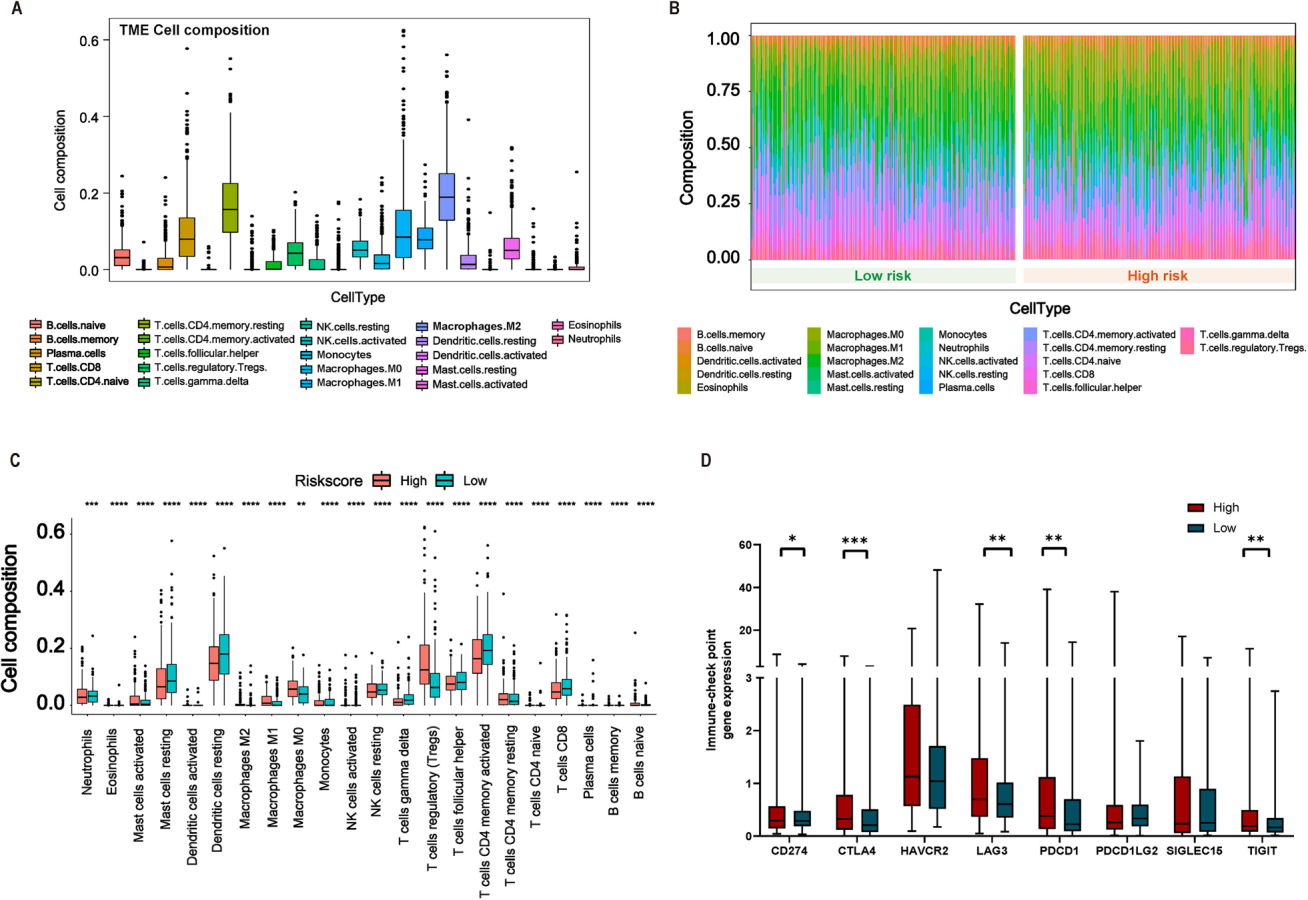
Correlation between immune infiltration level and Rho GTPase-related gene signature. **A** Immune cell composition in the Tumor microenvironment of HCC patients from TCGA cohort. **B** Immune cell composition profiling between high- and low-RGPRG score HCC patients. **C** Boxplot of immune cell composition between high- and low-RGPRG score HCC patients. **D** Immune checkpoint gene expression between high- and low-RGPRG score HCC patients. *P < 0.05, **P < 0.01, ***P <0.001, ****P < 0.0001

Considering the close relationship between RGPRG score and immune activity, we sought to further explore the immune infiltrate landscape of two RGPRG score groups. The 22 immune cell composition of HCC patients from the TCGA cohort was shown in Fig. 5A. CD8 T cells, resting memory CD4 T cells, M0 Macrophages, M1 Macrophages, and M2 Macrophages were the main immune cell composition of HCC patients. Many differential immune cell infiltrations could be observed between high- and low-RGPRG score groups (Fig. 5B). Patients with high RGPRG score was characterized by the high infiltration of resting memory CD4 T cells, M0 Macrophages, T cells regulatory (Tregs), naïve B cells, memory B cells, Plasma cells, naive CD4 T cells, Eosinophils, indicating a status of immunosuppression in this groups. CD8 T cells, activated memory CD4 T cells, activated NK cells, M1 Macrophages, M2 Macrophages, follicular helper T cells, gamma delta T cells, Neutrophils, resting Dendritic cells, activated Dendritic cells, resting NK cells, Monocytes, resting Mast cells, and activated Mast cells were abundant in the patients with low RGPRG score (Fig. 5C), suggesting enhanced antitumor environment. In addition, the expression of 8 classical immune checkpoint genes between two RGPRG score groups was further estimated in the TCGA cohort, and we found that Patients with high RGPRG score had higher expression levels of CD274, CTLA4, LAG3, PDCD1, and TIGIT (Fig. 5D), which presented another characteristic of immunosuppression.

### Single-cell analysis revealed the correlation between Rho GTPase phenotypes and immune tumor microenvironment heterogeneity

**Fig. 6:**
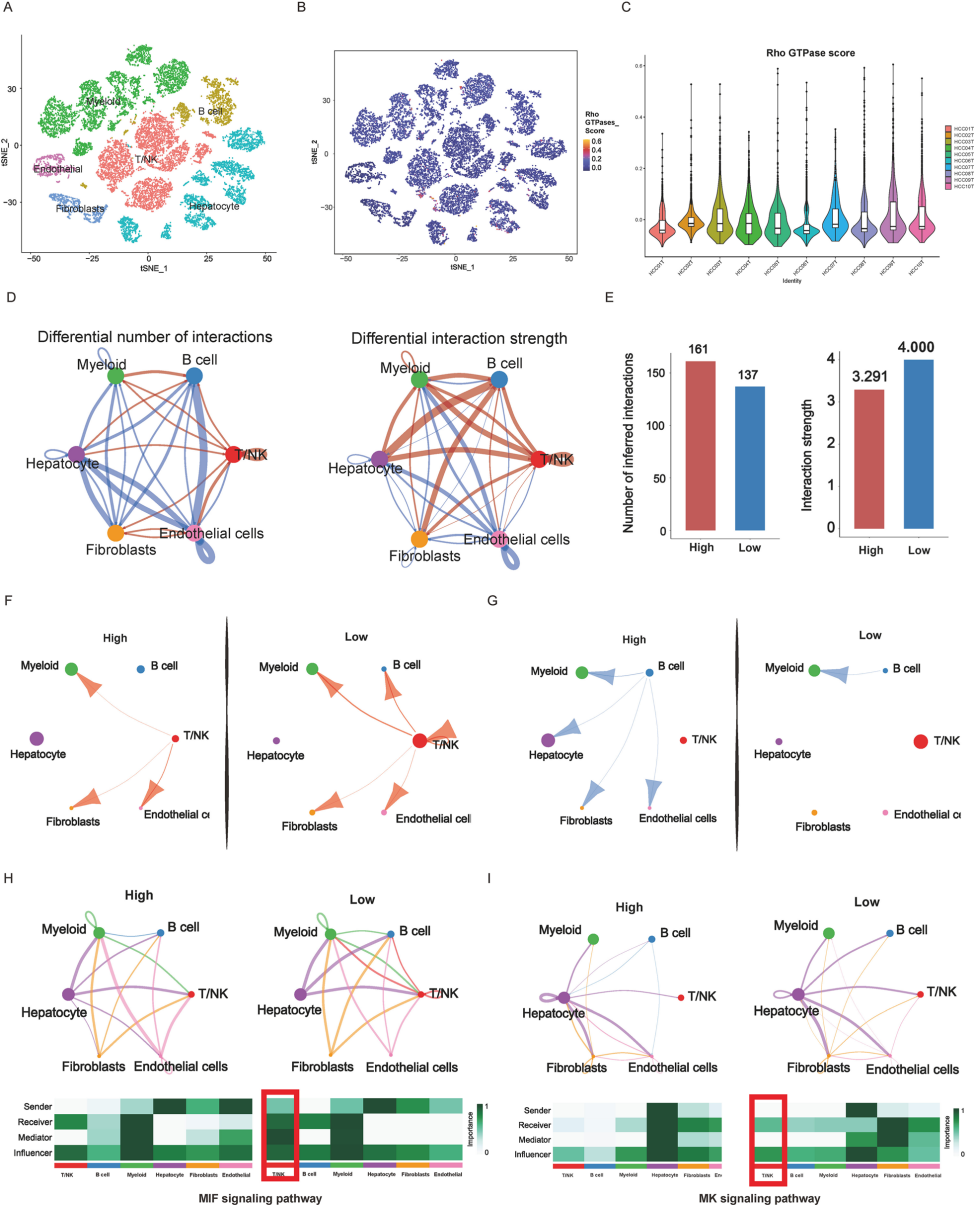
Single-cell cohort of HCC patients showing low-RGPRG score HCC patients possess an immune-active state. **A** t-SNE plot of the seven cell clusters derived from HCC samples. **B** t-SNE plot showing the distribution of RGPRG score in seven cell clusters. **C** Boxplot of RGPRG score in ten HCC samples. **D** Differential number of interactions (left) and strength (right) of seven cell clusters between high- and low-RGPRG score. **E** Number of inferred interactions (left) and strength (right) of seven cell clusters between high- and low-RGPRG score. **F** Circos plots of the putative ligand-receptor interactions between TNK cell and other cell clusters. Each brand points to interacting cell cluster. **G** Circos plots of the putative ligand-receptor interactions between B cell and other cell clusters. Each brand points to an interacting cell cluster. **H** Signaling pathway circle plots and network showing MIF signaling pathway between high- and low-RGPRG score groups. **I** Signaling pathway circle plots and network showing MK signaling pathway between high- and low-RGPRG score groups

We next sought to explore the effect of cell composition heterogeneity on Rho GTPase phenotypes at the single-cell level. 10 HCC samples were obtained with a total of 34,414 cells from the GSE149614 dataset. After quality control and standardization (Additional file 1: Fig. S6A, B), filtered cells were identified as 24 cell clusters (Additional file 1: Fig. S6C) and then annotated into 6 major clusters, including T/NK cells, B cells, fibroblasts, hepatocyte, myeloid cells, and endothelial cells with canonical marker genes as previously described [26] (Fig. 6A; Additional file 1: Fig. S6D, E). Using 16 Rho GTPase-signaling genes, the RGPRG score of each cell was presented in a t-Distributed Stochastic Neighbor Embedding (tSNE) plot, and we noticed that myeloid cells, the immunosuppression cells, showed higher RGPRG score than other cells, which may contribute to the generation of immunosuppressive tumor microenvironment (Fig. 6B; Additional file 1: Fig. S6F). Based on the distribution of RGPRG score, we could divide 10 HCC samples into high and low RGPRG score groups (Fig. 6C). Consistent with previous results [27], higher T cytotoxic scores were observed in HCC samples with low RGPRG score, but there was no significant difference between high- and low RGPRG score groups (P = 0.0767, Additional file 1: Fig. S7A). To further investigate the key players in the immune tumor microenvironment that contributed to Rho GTPase subtypes, we analyzed the cell-to-cell interactions and strength (Fig. 6D). Although the number of inferred interactions of the high RGPRG score group was slightly increased than the low RGPRG score group, enhanced interaction strength could be observed in the low RGPRG score group (Fig. 6E; Additional file 1: Fig. S7B). Interestingly, we noticed that T/NK cells displayed strong interaction strength with other cell types especially B cells and myeloid cells in low RGPRG score group (Fig. 6F), while in the high RGPRG score group, B cells had widespread communication with other cell types such as fibroblasts, hepatocyte, and endothelial cells except for T/NK cells (Fig. 6G). Additionally, we found that T/NK cells enhanced their communication with other cell types through MIF and MK signaling pathways in the low RGPRG score group (Fig. 6H, I). Ligand-Receptor Pairs analysis further displayed that enhanced communication between T/K cells and myeloid cells though MIF pathway (CD74-CXCR4), and MIF pathway (CD74 + CD44) in the patients with low RGPRG score, which identifying the increased recruitment of T/NK cell by macrophages in the low RGPRG score (Additional file 1: Fig. S7C). Collectively, these results further confirmed the immune tumor microenvironment heterogeneity between Rho GTPase subtypes and indicated that the low RGPRG score group had an immune-active state tumor microenvironment.

### The RGPRG score model can predict the efficacy of immunotherapy

**Fig. 7:**
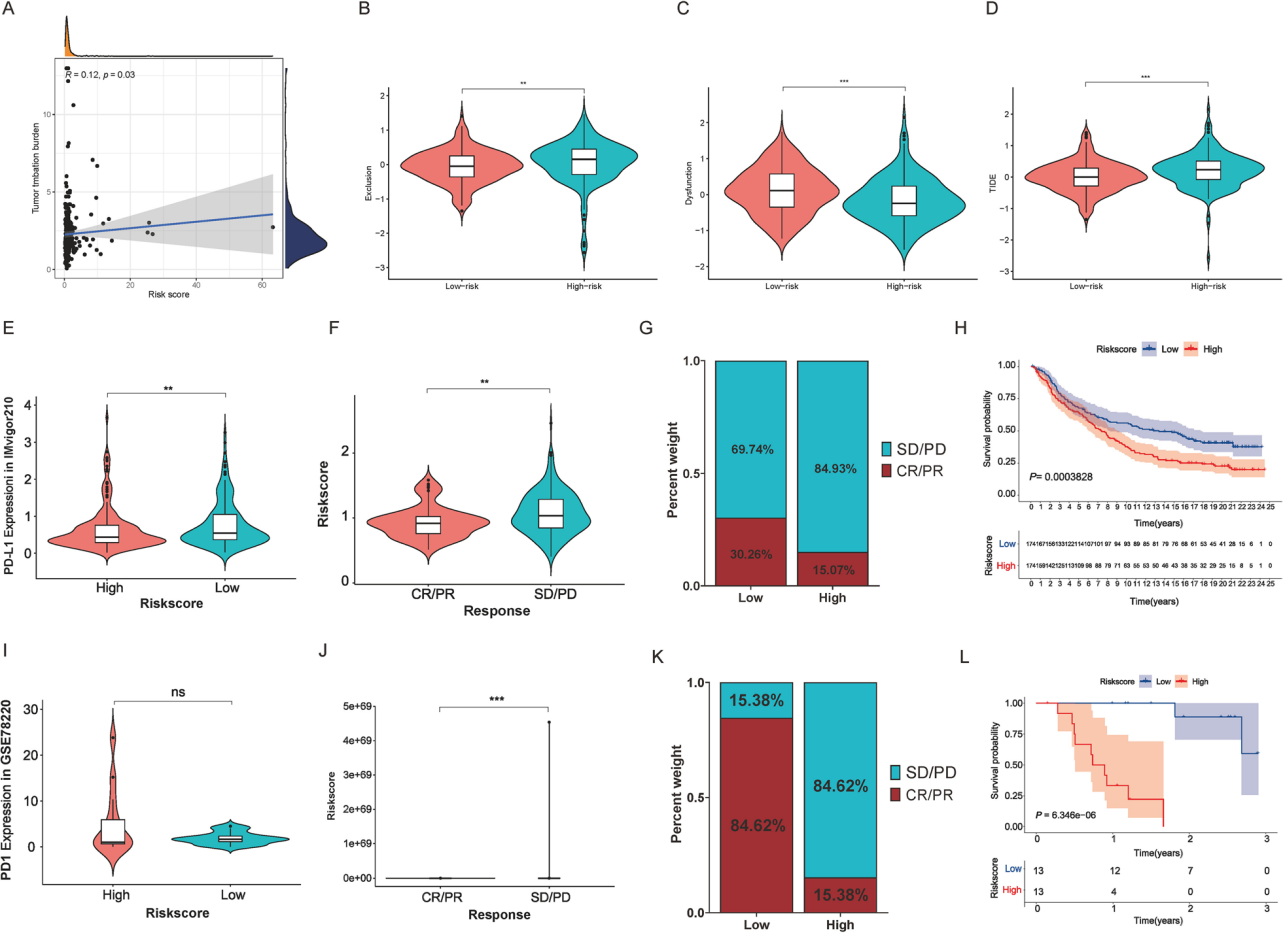
Rho GTPase-related gene signature can predict efficacy of immunotherapy. **A** Scatterplots displaying the correlation of RGPRG score and tumor mutation burden. **B** The relationship of Exclusion between high- and low-RGPRG score HCC patients. **C** The relationship of Dysfunction between high- and low-RGPRG score HCC patients. **D** The relationship of TIDE between high- and low-RGPRG score HCC patients. **E**, **I** The boxplot shows the correlation of PDL1 expression and RGPRG score groups in IMvigor210, and the differences in PD1 expression between RGPRG score groups in GSE78220. **F**, **J** Distinct clinical outcomes of anti-PDL1 and PD-1 blockade immunotherapy between high- and low-RGPRG score groups in IMvigor210 and GSE78220. CR, complete response; PR, partial response; SD, stable disease; PD, progressive disease. **G**, **K** The proportion of distinct clinical outcomes between high- and low-RGPRG score groups in IMvigor210 and GSE78220. **H**, **L** Kaplan–Meier survival curve showing the survival difference between high- and low-RGPRG score groups in IMvigor210 and GSE78220

Considering the core role of Rho GTPase phenotypes in the immune tumor microenvironment, we applied the TIDE database to further predict the immune escape of tumor cells. Tumor mutation burden (TMB) (Fig. 7A), Exclusion (Fig. 7B), and TIDE (Fig. 7D) scores were significantly high in the high RGPRG score group, whereas Dysfunction scores (Fig. 7C) were opposite, suggesting that a lower response rate to immunotherapy in high RGPRG score group. Furthermore, we selected an anti-PD-L1 treated cohort (IMvigor210) and an anti-PD-1 treated cohort (GSE78220) to assess the response rate to immune checkpoint inhibitor (ICI) treatment. We observed that PD-L1 expression in the low RGPRG score group from IMvigor210 was significantly higher than that of the high RGPRG score group (*P* < 0.01, Fig. 7E), while there was no difference between PD-1 expression and RGPRG score group in GSE78220 (Fig. 7I). Patients with low RGPRG score exhibited significant clinical benefits (Fig. 7F, G, J, K) and markedly prolong their survival compared with those patients with high RGPRG score (Fig. 7H, L). These analyses result indicates that the RGPRG score model had potential predictive value for anti-PD-L1 and anti-PD-1 immunotherapy and guide ICI treatment option.

### The RGPRG score model predicts potential targeted drugs for HCC patients

**Fig. 8:**
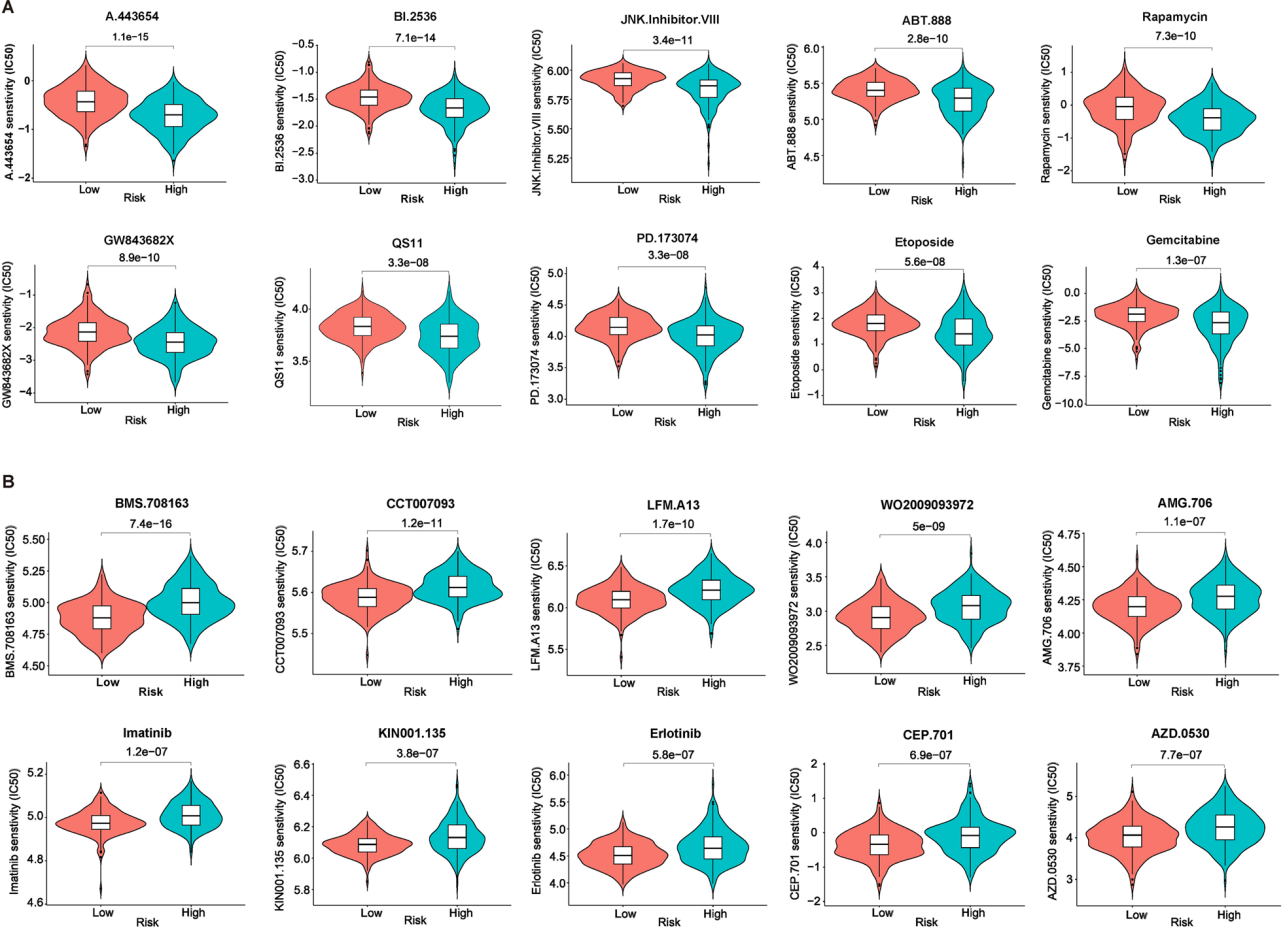
Rho GTPase-related gene signature can guide the selection of chemotherapeutic Agents. **A** Top 10 drugs are more sensitive for high-RGPRG score patients. **B** Top 10 drugs are more sensitive for low-RGPRG score patients

To further explore the correlation between drug resistance and Rho GTPase phenotypes, we applied the GDSC database to assess the response of Rho GTPase subtypes on various drugs. The IC50 values of 91 potential drugs exhibit differences between high and low RGPRG score groups, suggesting a potential relationship between RGPRG scores and drug sensitivity or resistance (Additional file 1: Fig. S8). According to P value, the top 10 ranked drugs including A.443654, BI.2536, JNK Inhibitor VIII, ABT.888, Rapamycin, GW843682X, QS11, PD.173074, Etoposide, and Gemcitabine displayed more drug sensitivity to high RGPRG score patients (Fig. 8A), whereas another top 10 ranked drugs including BMS.708163, CCT007093, LFM.A13, WO2009093972, AMG.706, Imatinib, KIN001.135, Erlotinib, CEP.701, and AZD.0530 showed more sensitivity to low RGPRG score patients (Fig. 8B). These results confirmed the strong correlation between drug resistance and Rho GTPase phenotypes, which further guide tumor therapeutic drug selection. **P < 0.01, ***P < 0.001

### The RGPRG score model has extensive prognostic value in pan-cancer

Based on the pan-cancer cohorts from TCGA database, we conducted a pan-cancer survival analysis to further evaluate the extensive prognostic value of Rho GTPase phenotypes. The distribution of RGPRG score in 35 different cancers was presented in Additional file 1: Fig. S9A, and we noticed that Cervical Adenosquamous (CSAC), esophageal squamous cell carcinoma (ESCC), Testicular Germ Cell Tumors (TGCT), and Kidney Chromophobe (KICH) had higher RGPRG score, while uterine carcinosarcoma (USC) and Lymphoid Neoplasm Diffuse Large B-cell Lymphoma (DLBC) had lower RGPRG score than other cancer types. Significant survival differences between high and low RGPRG score groups could be observed in almost 27 cancer types (Additional file 1: Fig. S9B). Notably, patients with low RGPRG score always exhibited better clinical outcome than those patients with high RGPRG score in pan-cancer, and this result further confirmed the excellent prognostic robustness and extensive prognostic value of the RGPRG score model.

### Key Rho GTPase-signaling gene SFN may become the potential target for the treatment of HCC

**Fig. 9:**
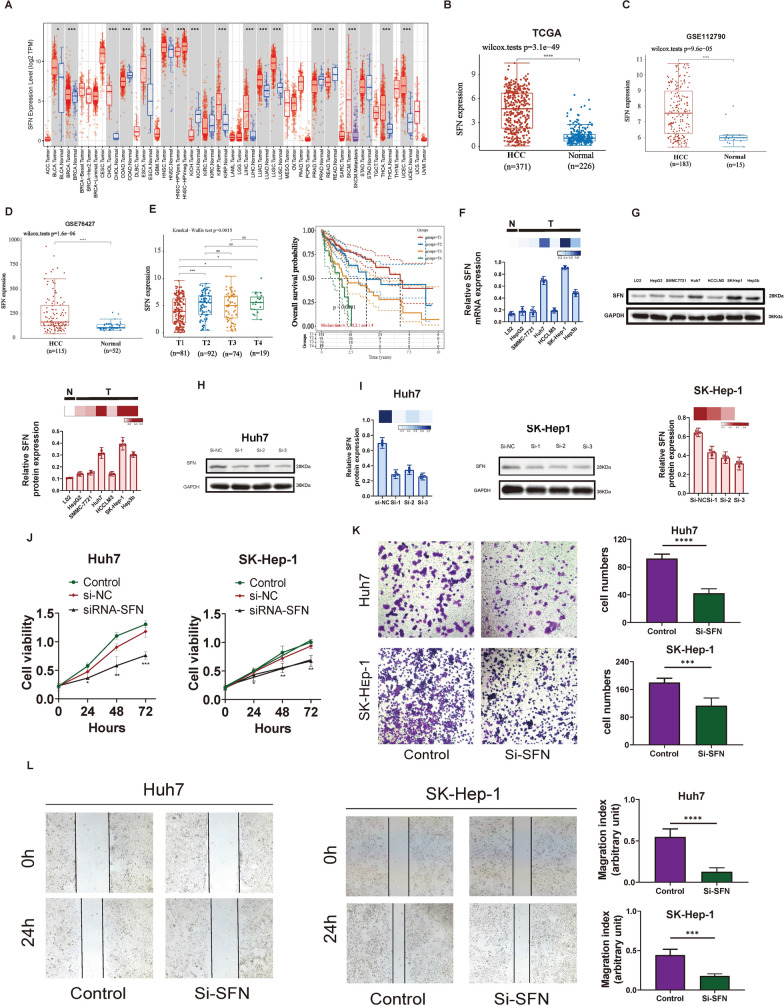
SFN could be the potential target in the treatment of HCC. **A** SFN expression level in pan-cancer. **B**–**D** SFN was significantly highly expressed in HCC samples than that of the normal samples based on (**B**) TCGA database, (**C**) GSE112790 cohort, and (**D**) GSE76427 cohort. **E** SFN expression was associated with the TNM stage of patients with HCC, and HCC patients with T1 had better survival than those patients with T2, T3, and T4. **F** The relative expression of SFN in seven HCC cell lines at mRNA level. **G** The relative expression of SFN in seven HCC cell lines at the protein level. **H** Knockdown efficiency of SFN in Huh7 cell line. **I** Knockdown efficiency of SFN in Sk-Hep-1 cell line. **J** Knockdown of SFN could significantly inhibit cell proliferation in the Huh7 cell line and Sk-Hep-1 cell line. **K**, **L** Downregulation of SFN in Huh7 and SK-Hep-1 cell lines inhibited cell invasion and migration. *P < 0.05, **P < 0.01, ***P < 0.001, ****P < 0.0001

Among 16 Rho GTPase-signaling signature genes, ARHGAP11A, IQGAP3 KIF18A, and SFN were independent prognostic genes based on the above multivariate logistic regression result. To further retain biologically significant and potentially functional differentially expressed genes, we identified key Rho GTPase-signaling genes based on higher |log2FC| values (|log2FC|> 1) and lower p values (p < 0.01) in the Gene Expression Profiling Interactive Analysis (GEPIA) database. The result displayed that only SFN was significantly overexpressed in HCC samples (from TCGA database) than normal samples (match TCGA normal and Genotype-Tissue Expression (GTEx) data) (Additional file 1: Fig. S10A), and thus we selected SFN for further analysis. We observed the expression level of SFN in pan-cancer and found that SFN were highly expressed in 12 cancer types (Fig. 9A). In three different cohorts from TCGA database, GSE112790, and GSE76427 cohorts, SFN also exhibited significantly higher expression in HCC samples than in normal samples (Fig. 9B–D). In addition, there was a significant difference between SFN expression level and different T stage of HCC patients, and early T-stages (T1 and T2) had better survival than advanced T stages (T3 and T4) (Fig. 9E). To explore the function of SFN expression in HCC, we first compared the expression level of SFN in 7 HCC cell lines and the results showed that SFN mRNA (Fig. 9F) and protein level (Fig. 9G) in Huh7, Sk-Hep-1, and Hep3B cell lines were the highest compared with the L02 cells. We selected Huh7, Sk-Hep-1 cell line for further function analysis, and the siRNA plasmid was constructed to knock down SFN expression in these two cell lines. The result of the western blot implied that SFN expression in the Huh7 cell and Sk-Hep-1 cell could be both significantly downregulated in the Si-3 group (Fig. 9H–I). CCK-8 assay demonstrated that knocking down SFN could significantly inhibit Huh7 and Sk-Hep-1 cells’ proliferation rate (Fig. 9J), suggesting that SFN may play a vital role in the progression of HCC cells. In addition, transwell assay and wound healing assay proved that down-regulated SFN could suppress the migratory and invasive capability in Huh7 and SK-Hep-1 cells (Fig. 9K–L). Furthermore, we conducted GSEA analysis on SFN expression, and we noticed that metabolism-related pathways such as metabolism cytochrome p450, fatty acid metabolism, glycine serine, and threonine metabolism, retinol metabolism, and PPAR signaling pathway (Additional file 1: Fig. S10B), which contributed to Rho GTPase activities.

## Discussion

Comprehensive transcriptomic analysis of Rho GTPase signaling-related DEGs in HCC may broaden our knowledge of the molecular events relevant to this highly aggressive malignancy [28–30]. Herein, we classified HCC patients into two clusters including cluster 1 and cluster 2 based on DEGs of the Rho GTPase patterns, which were correlated with the prognosis and progression of advanced HCC. A high-sensitivity prognostic model named RGPRG score system was then proposed, and this model enables evaluation of pan-cancer prognosis including HCC with higher accuracy, implying its wide application. Furthermore, a high RGPRG score was found to be significantly associated with poor prognosis, high TMB, and increased immunosuppressive cell infiltration, while a low RGPRG score was correlated with the immune-active state and clinical benefit of immunotherapy. Previous studies have linked Rho GTPase to chemoresistance [31], while our work also found the 91 potential targeted drugs for HCC patients with high and low RGPRG score, assisting in the guidance of personalized medication. Therefore, Rho GTPase in fatal malignancy, especially in HCC, progression, and prognosis still need more attention.

There are several important findings in our work, Firstly, our work performed the first transcriptomic characterization of Rho GTPase signaling-related genes in HCC, and new HCC molecular subtypes were identified. Based on the expression of 67 identified Rho GTPase signaling-related DEGs, HCC patients were obviously divided into two subtypes. Patients in cluster 2 were characterized by the higher expression of Rho GTPase-related genes (denoted as Rho GTPase activation subgroup), while cluster 1 was featured by lower expression of Rho GTPase-related genes (denoted as Rho GTPase inactivation subgroup). Rho GTPase activation subgroup had poor overall survival probability, which may attribute to the ability of Rho GTPase activation to promote cancer cell invasiveness [30]. Further clinical characteristics analysis demonstrated that there was a higher proportion of TNM stage III, Grade 3 in cluster 2, suggesting a strong correlation between Rho GTPase subtypes with advanced malignant HCC and poor prognosis. Therefore, the Rho GTPase subtypes can be used as a novel prognostic subtype to evaluate the progression of HCC. Of note, previous studies also led to a similar 2-subgroup allocation based on the biological function [32, 33], which supports the reliable molecular subtype procedure in our study.

Second, compared to traditional TNM stage and other published prediction models [20–23, 34], we developed a 16-gene prognostic model with a better and more robust predictive accuracy (most of AUC value > 0.8). These sixteen genes collectively contribute to HCC biology by either promoting cancer development or inhibiting tumor growth, depending on their specific functions. Among them, we select SFN (also known as 14–3-3 sigma) for further in vitro experiment. Both SFN and Rho GTPases regulate cell cytoskeleton remodeling and cell migration, which suggests a possible interaction between the signaling pathways regulated by these two groups of proteins. Emerging evidence supports the notion of mutual regulation between 14-3-3 sigma and Rho GTPases. The localized activation of Rho family GTPases, stimulated by the cell microenvironment, leads to the formation of discreet actin structures that either promote or inhibit cell migration [35–38]. 14-3-3σ was found to regulate actin dynamics of tumor cell in a ligand-binding mechanism, which provides a signaling switch bridging cytoskeletal dynamic equilibrium with cell motility [39]. In addition, a number of studies have highlighted different mechanisms by which 14-3-3 family members regulate actin dynamics either through stabilizing cofilin phosphorylation or by inhibiting signals through the AKT-RhoA pathway [40–43]. Our in vitro experiment also confirmed that down-regulated SFN could suppress the migratory and invasive capability in HCC cell lines, suggesting its potential link between Rho-GTPase signaling and SFN. Taken together, the collective evidence from these studies strongly supports the biological plausibility of the identified genes in HCC, underscoring their potential as valuable targets for further investigation in the quest for improved diagnostics and therapeutic strategies for this challenging disease.

Moreover, we applied DCA, a method that can assist physicians in selecting the most appropriate clinical prediction model when comparing multiple models. 16-gene prognostic model show a better clinical utility compared with other published model such as pyroptosis-related risk model [20], ferroptosis-related risk model [21], cuproptosis-related risk model [23], and immune-related risk model [22]. By evaluating the RGPRG score in HCC patients, clinicians and researchers can gain insights into the potential aggressiveness of the tumor and the likelihood of disease progression, which can help guide treatment decisions, prognosis assessment, and the development of targeted therapies in HCC. Furthermore, the 16-gene prognostic model can expand to predict 27 cancer survival, showing an extensive prognostic value in multiple cancer types. Single and multiple Cox regression analyses identified that RGPRG score was an independent prognostic factor. Of note, the result of GSEA analysis indicated that the high RGPRG score group was mainly enriched in pathways related to cell primary immunodeficiency, which may attribute to the existence of several signature genes related to immune function (CYBB [44], ECT2 [45], PRC1 [46], SFN [47]).

Thirdly, we further found a strong connection between RGPRG score and TME features. Different immune cell has different roles. For instance, T cell regulatory (Tregs) is a key player in immune evasion and tumor growth, which can counteract T cell-mediated immune response [48]. Tumor-associated macrophages could be classified into three subtypes including M0 (undifferentiated), M1 (anti-tumor), and M2 (tumor-promoting) by their transcriptional signatures [49]. Of note, a previous study also demonstrated that M0 macrophages appear to promote cancer cell growth at high concentrations, while M1 types are the opposite [50]. CD8 T cells and activated NK cells have long been reported to kill cancer cells directly and induce the activation of different immune cells [51, 52]. T-follicular helper cells are a subset of CD4 + T cells that play a vital role in protective immunity helping B cells produce effective humoral immune responses [53]. Another immune-active cell named activated Dendritic cells leads to the activation of cytotoxic T cells, which could induce antigen-specific immune responses and kill cancer cells eventually [54]. In our study, the deconvolution algorithm CIBERSORT revealed that multiple tumor immunosuppression subsets are significantly increased in the high RGPRG score group, including T cells regulatory (Tregs) and M0 macrophages. On the other hand, anti-tumor immunity subsets such as CD8 T cells, activated NK cells, T-follicular helper cells, and activated Dendritic cells were significantly enriched in the low RGPRG score group. Therefore, low RGPRG score group associates with immune activation while high RGPRG score group associates with immunosuppression, which accounts for their clinical outcome between different Rho GTPase phenotypes.

Fourthly, we using single-cell cohort to shed light on the phenomenon of immune tumor microenvironment heterogeneity between two Rho GTPase phenotypes. We observed that T/NK cells enhance intercellular communication, especially with myeloid cells through MIF pathways in the low RGPRG score group. T cells and NK cells have previously been reported to display robust cytotoxic activity and serve an immuno-regulatory role [55], and their activation associates with MIF pathway [56]. On the other hand, extracellular MIF is necessary for steady-state activation of Rho GTPase members, resulting in tumor invasion and metastasis [57]. Altogether, Rho GTPase may bridge HCC and immunocytes infiltration to affect HCC invasion and metastasis.

Lastly, we explored the role of RGPRG score in predicting response to immunotherapy and chemotherapy. We showed that a high RGPRG score was correlated with higher TMB, increased TIDE score, lower PD-L1 expression, and a low response to ICI treatment, which indirectly indicated that RGPRG score could help clinicians forecast the response to ICI, and match patients most likely to benefit from immunotherapy treatment. Apart from immunotherapy, potential chemotherapeutic drugs for patients with high RGPRG score are also predicted using the GDSC database. Rapamycin, an inhibitor of mTOR, can block HCC progression triggered by p53 and Tsc1 insufficiency [58]. Etoposide and Gemcitabine are common chemotherapeutic agents for HCC that have been proven to exhibit their anti-tumoral effects by induction of cell cycle arrest in S or G2 [59, 60]. However, Others such as A.443654, BI.2536, JNK Inhibitor VIII, ABT.888, GW843682X, QS11, and PD.173074 have not been previously reported, requiring further explore their association with HCC progression or Rho GTPase.

Some limitations also exist in our study that is worth noting. Although we have used multiple cohorts to validate the accuracy of RGPRG score model, prospective cohorts are still needed to prove the clinical reliability of this prognostic model. In addition, we revealed that HCC immune infiltration might correlate with Rho GTPase phenotypes using transcriptomics and single-cell data, but the biological mechanisms behind these phenomenons remain clear investigation. More importantly, although we verify the expression and function of key Rho GTPase-related gene SFN in HCC cell lines, more research should focus on these 16 signature genes and their detailed mechanisms on Rho GTPase regulation and the progression of HCC.

In conclusion, our current work proposed a high-sensitivity prognostic model named RGPRG score system, and revealed that a high RGPRG score is an independent prognostic factor of HCC that is associated with advanced disease stage, poor prognosis, increased immunosuppressive cell infiltration, and a low response to immunotherapy. Our work may provide additional clinical insights underlying biological features of Rho GTPase in HCC.

### Supplementary Information

**Additional file 1:** **Figure S1.** Association between TNM stage and survival in the three HCC cohorts.** Figure S2. **Identifying candidate Rho GTPase-related genes in HCC patients.** Figure S3. **Distribution of RGPRG score and survival status with increasing RGPRG score in HCC patients. **Figure S4. **Biofunction analysis of Rho GTPase-related gene signature.** Figure S5. **Gene mutation of the Rho GTPase-related gene signature.** Figure S6.** Quality control and cell type characterization in GSE14961 cohort. (A) Data quality control (QC) of single-cell RNA-seq data from GSE149614 cohort. HB, hemoglobin; MT; mitochondria. (B)The association between nCount RNA and four QC parameters. With the increased nCount RNA, the small number of the mitochondrial and ribosome content, the better the activity of the cells. (C) The tSNE plot of identified 24 cell clusters from 10 HCC patients. (D) The Dotplot showing the average expression levels of canonical marker genes of six major cell types in 24 cell clusters. (E) The tSNE plots of the expression levels of marker genes of six major cell types. (F) The boxplot showing the distribution of RGPRG score in six major cell types. **Figure S7.** T cytotoxic score, interactions, and Ligand-Receptor pairs between two RGPRG score groups. (A) Differences in T cytotoxic score between two RGPRG score groups. (B) Differences in number of interactions and interaction weight/strength between two RGPRG score groups. (C) Comparison of the ligand-receptor pairs between two RGPRG score groups. **Figure S8.** Drug sensitivity analysis based on RGPRG score in HCC patients. (A) 38 drugs were more sensitive to HCC patients with low RGPRG score. (B) 53 drugs were more sensitive to HCC patients with high RGPRG score. **Figure S9. **Rho GTPase-related gene signature can predict pan-cancer prognosis. **Figure S10.** Expression profiling and GSEA analysis of key RHO-GTPase genes. (A) ARHGAP11A, IQGAP3 KIF18A, and SFN expression level between LIHC and normal samples from GEPIA database. LIHC: Liver Hepatocellular Carcinoma. (B) GSEA analysis of patients with low expression SFN.

**Additional file 2: Table S1.** Rho GTPase-signaling gene set. **Table S2.** 67 identified Rho GTPase-signaling related differentially expressed genes between HCC and normal samples in TCGA cohort. **Table S3.** 180 identified Rho GTPase-signaling related prognostic genes in TCGA cohort. **Table S4.** Clinical Characteristics of a single-cell cohort of HCC samples (GSE149614).

## Data Availability

All data generated or analyzed during this study are included in this published article.
