# Supplementary material for: Adverse clinical outcomes and immunosuppressive microenvironment of RHO-GTPase activation pattern in hepatocellular carcinoma
Source: J Transl Med. 2024 Jan 31;22:122. doi: 10.1186/s12967-024-04926-0 (PMC10832138; doi:10.1186/s12967-024-04926-0)
Supplement: Supplementary file 1 — Additional file 1: Figure S1. Association between TNM stage and survival in the three HCC cohorts. Figure S2. Identifying candidate Rho GTPase-related genes in HCC patients. Figure S3. Distribution of RGPRG score and survival status with increasing RGPRG score in HCC patients. Figure S4. Biofunction analysis of Rho GTPase-related gene signature. Figure S5. Gene mutation of the Rho GTPase-related gene signature. Figure S6. Quality control and cell type characterization in GSE14961 cohort. (A) Data quality control (QC) of single-cell RNA-seq data from GSE149614 cohort. HB, hemoglobin; MT; mitochondria. (B)The association between nCount RNA and four QC parameters. With the increased nCount RNA, the small number of the mitochondrial and ribosome content, the better the activity of the cells. (C) The tSNE plot of identified 24 cell clusters from 10 HCC patients. (D) The Dotplot showing the average expression levels of canonical marker genes of six major cell types in 24 cell clusters. (E) The tSNE plots of the expression levels of marker genes of six major cell types. (F) The boxplot showing the distribution of RGPRG score in six major cell types. Figure S7. T cytotoxic score, interactions, and Ligand-Receptor pairs between two RGPRG score groups. (A) Differences in T cytotoxic score between two RGPRG score groups. (B) Differences in number of interactions and interaction weight/strength between two RGPRG score groups. (C) Comparison of the ligand-receptor pairs between two RGPRG score groups. Figure S8. Drug sensitivity analysis based on RGPRG score in HCC patients. (A) 38 drugs were more sensitive to HCC patients with low RGPRG score. (B) 53 drugs were more sensitive to HCC patients with high RGPRG score. Figure S9. Rho GTPase-related gene signature can predict pan-cancer prognosis. Figure S10. Expression profiling and GSEA analysis of key RHO-GTPase genes. (A) ARHGAP11A, IQGAP3 KIF18A, and SFN expression level between LIHC and normal samples from GEPIA database. LIHC: [file 12967_2024_4926_MOESM1_ESM.pdf]

Additional file 1

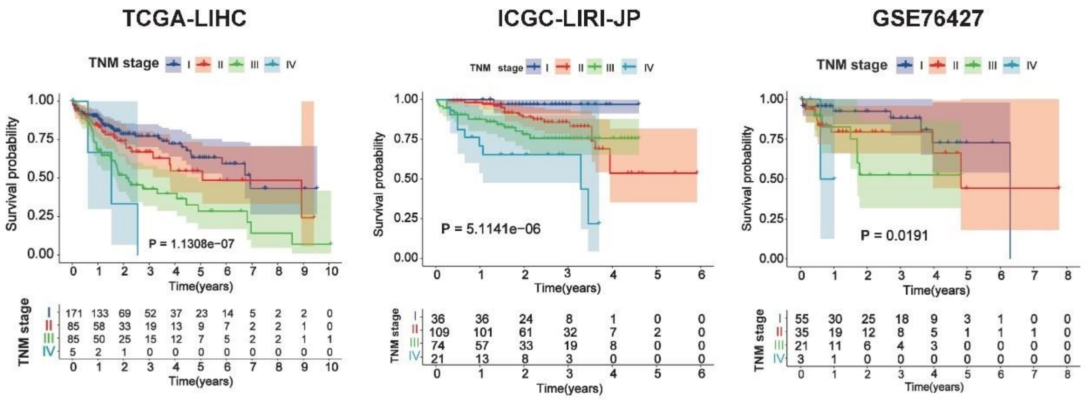

Fig. S1 Association between TNM stage and survival in the three HCC cohorts.

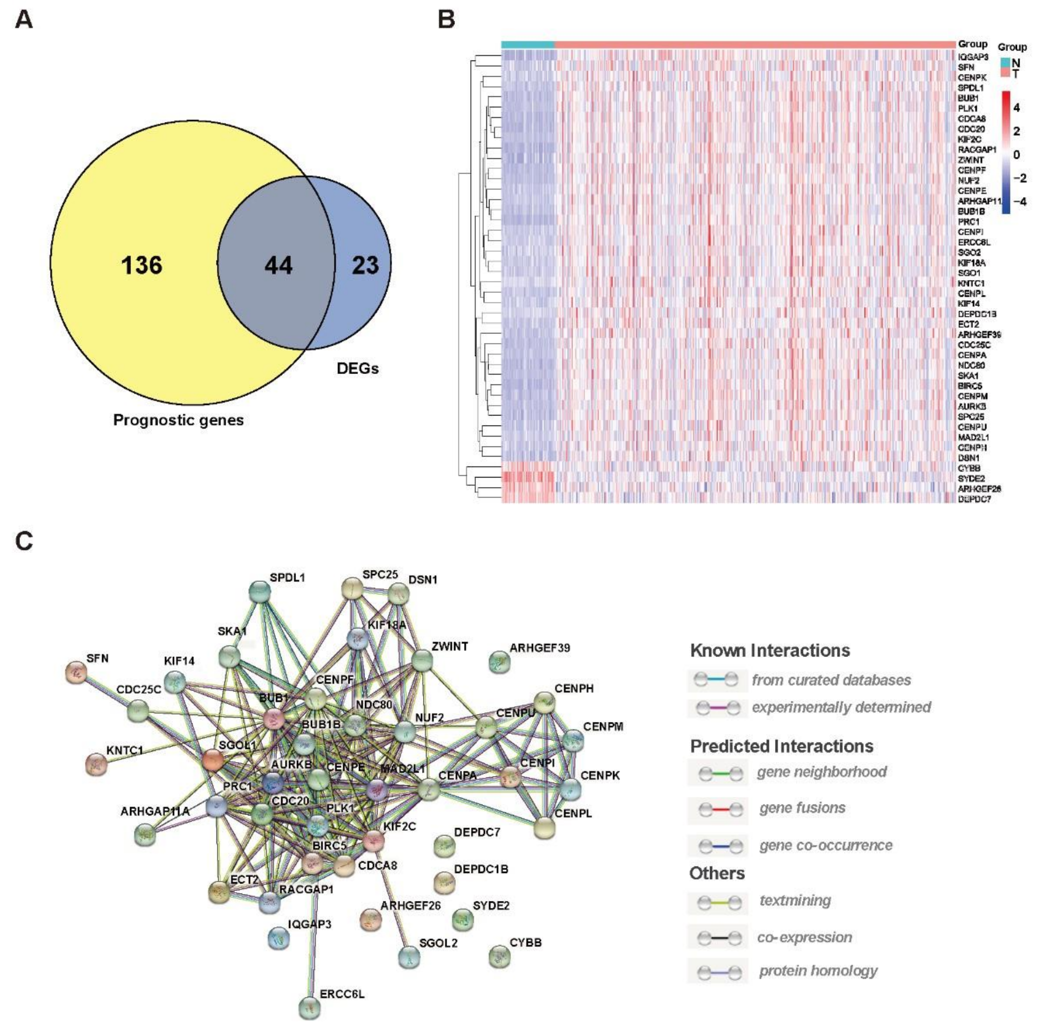

Fig. S2 Identifying candidate Rho GTPase-related genes in HCC patients. (A) Venn diagram identifying 44 Rho GTPase-related DEGs that correlated with prognosis in HCC patients. (B) A heatmap showing that 40 candidate genes were significantly upregulated and 4 candidate genes were significantly downregulated in HCC patients. (C) PPI network displaying the interactions among the 44 genes.

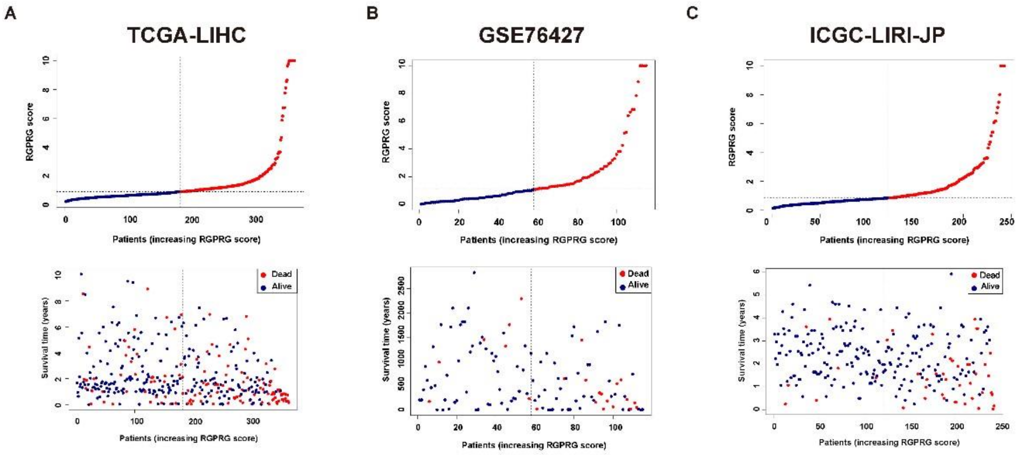

Fig. S3 Distribution of RGPRG score and survival status with increasing RGPRG score in HCC patients. (A, B, C) The distribution of RGPRG score in the HCC patients from the TCGA-LIHC, GSE76427, ICGCLIRI-JP cohorts (above). The scatter plot of the survival time and status with increasing RGPRG score of HCC patients from TCGA-LIHC, GSE76427, ICGC-LIRI-JP cohorts (below).

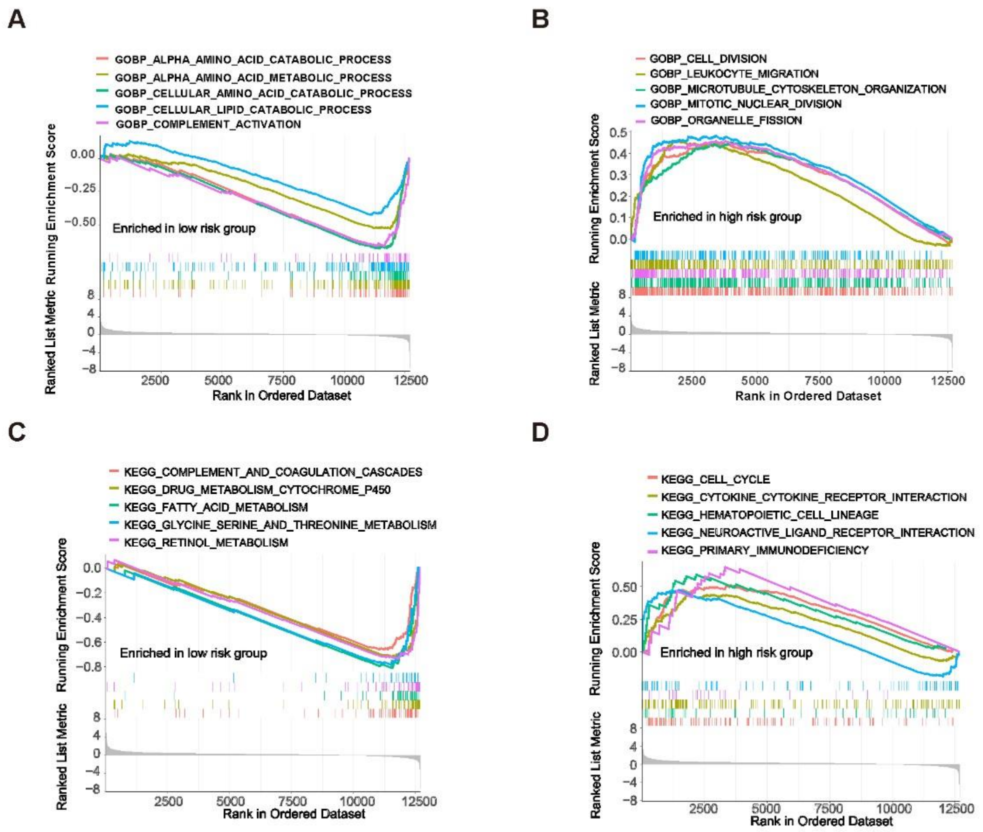

Fig. S4 Biofunction analysis of Rho GTPase-related gene signature. (A) Gene set enrichment analysis (GESA) showing the Gene Ontology (GO) terms for the patients in the low-RGPRG score group. (B) GESA shows the GO terms for the patients in the high-RGPRG score group. (C) GESA showing Kyoto Encyclopedia of Genes and Genomes (KEGG) pathway for the patients with the low-RGPRG score group. (D) GESA showing KEGG pathway for the patients with the high-RGPRG score group.

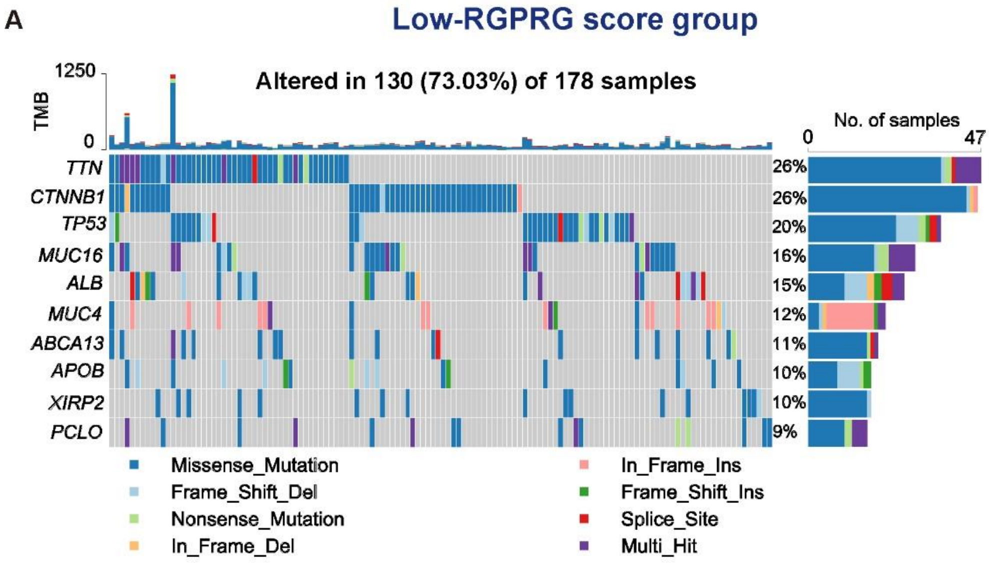

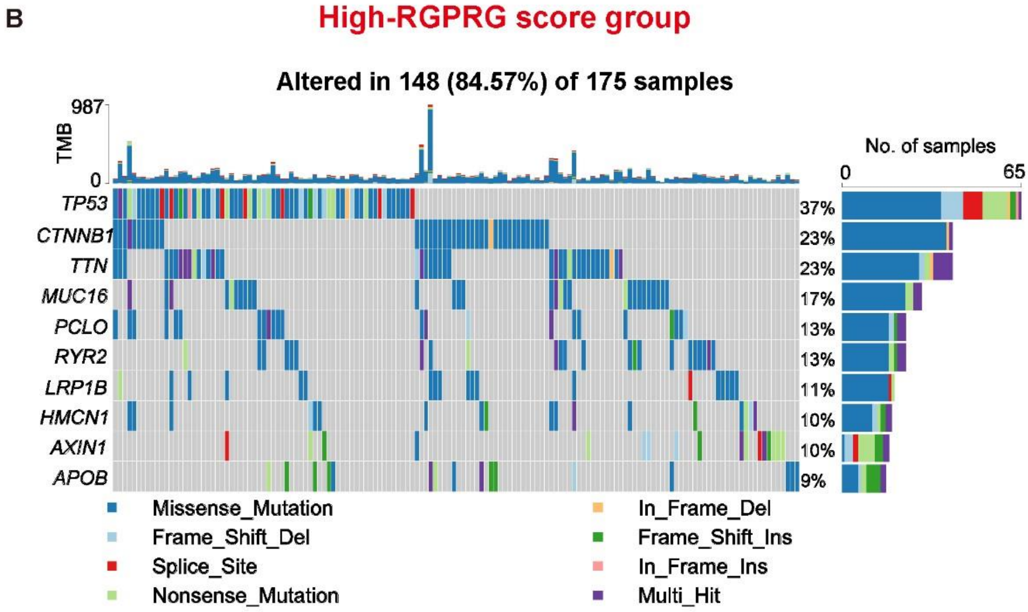

Fig. S5 Gene mutation of the Rho GTPase-related gene signature. (A) The mutation frequency and classification in the low-RGPRG score group. (B) The mutation frequency and classification in the high-RGPRG score group.

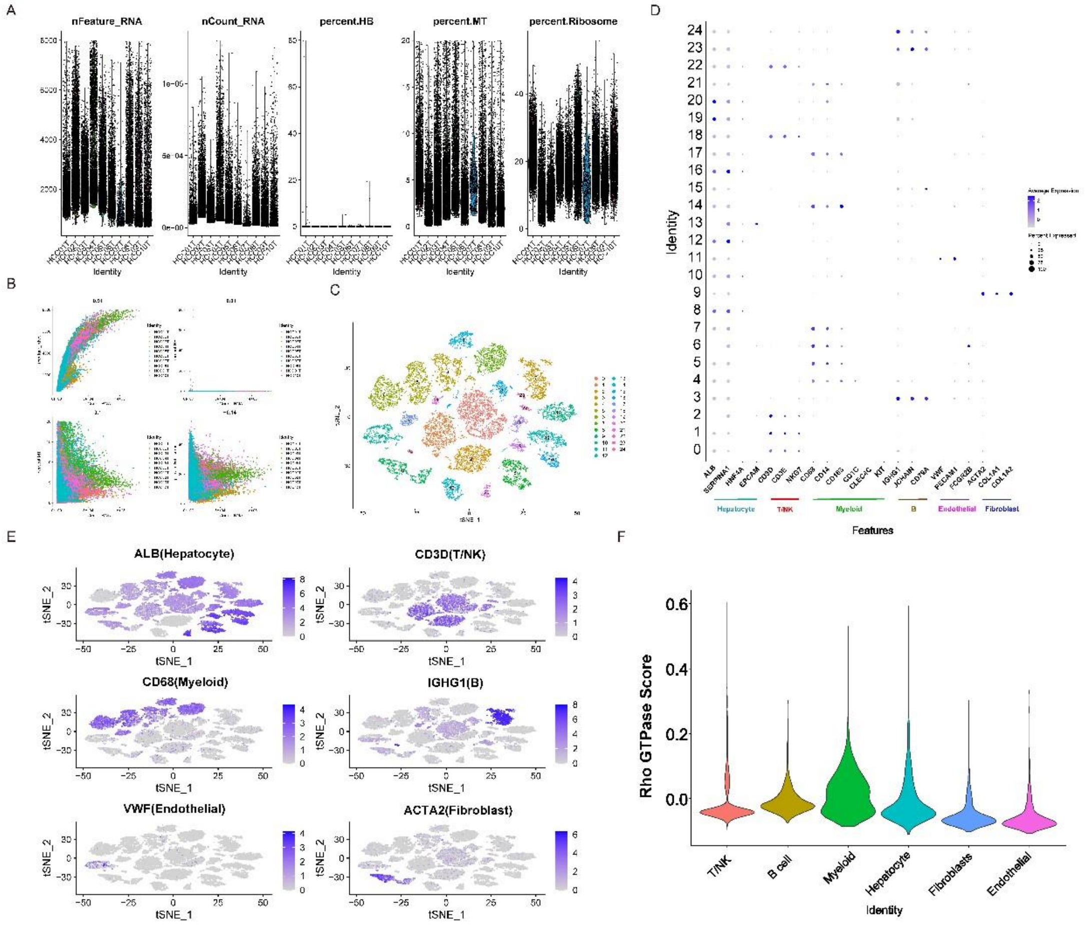

Figure S6. (A) Data quality control (QC) of single-cell RNA-seq data from GSE149614 cohort. HB, hemoglobin; MT; mitochondria. (B)The association between nCount RNA and four QC parameters. With the increased nCount RNA, the small number of the mitochondrial and ribosome content, the better the activity of the cells. (C) The tSNE plot of identified 24 cell clusters from 10 HCC patients. (D) The Dotplot showing the average expression levels of canonical marker genes of six major cell types in 24 cell clusters. (E) The tSNE plots of the expression levels of marker genes of six major cell types. (F) The boxplot showing the distribution of RGPRG score in six major cell types.

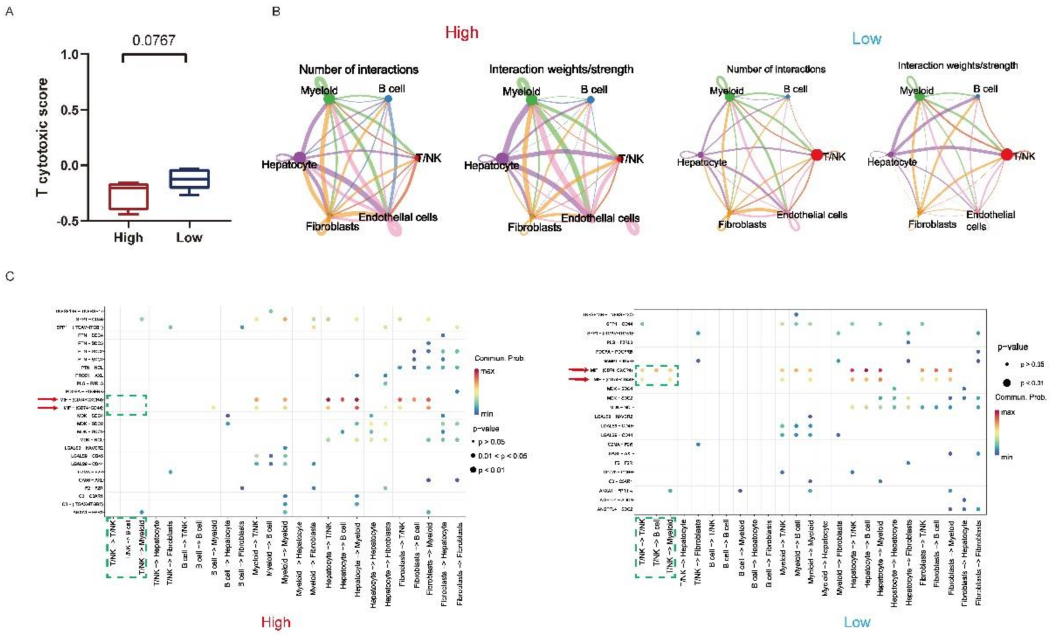

Figure S7. (A) Differences in T cytotoxic score between two RGPRG score groups. (B) Differences in number of interactions and interaction weight/strength between two RGPRG score groups. (C) Comparison of the ligand-receptor pairs between two RGPRG score groups.

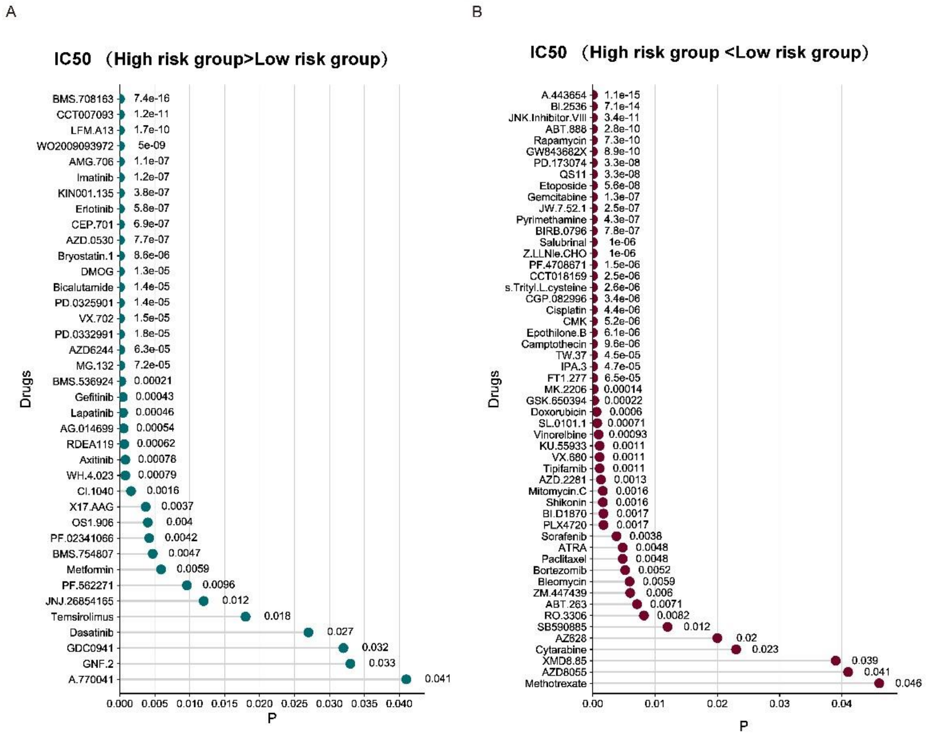

Figure S8. (A) 38 drugs were more sensitive to HCC patients with low RGPRG score. (B) 53 drugs were more sensitive to HCC patients with high RGPRG score.

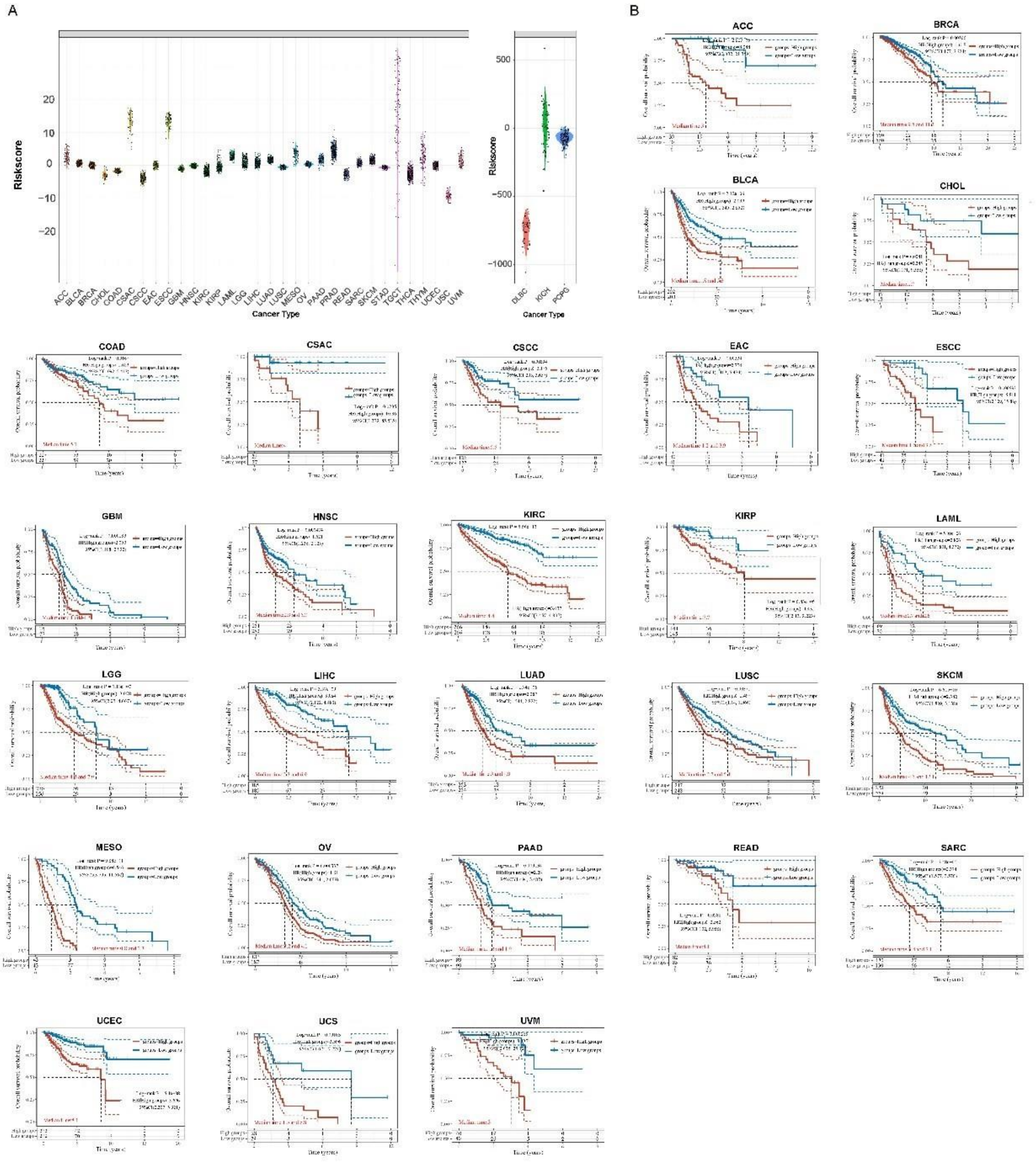

Figure S9. Rho GTPase-related gene signature can predict pan-cancer prognosis. (A) The distribution of RGPRG score in 35 cancer types. (B) Kaplan-Meier survival curve shows high-RGPRG score cancer patients of 27 different cancer types had significantly worse survival compared with low-RGPRG score cancer patients.

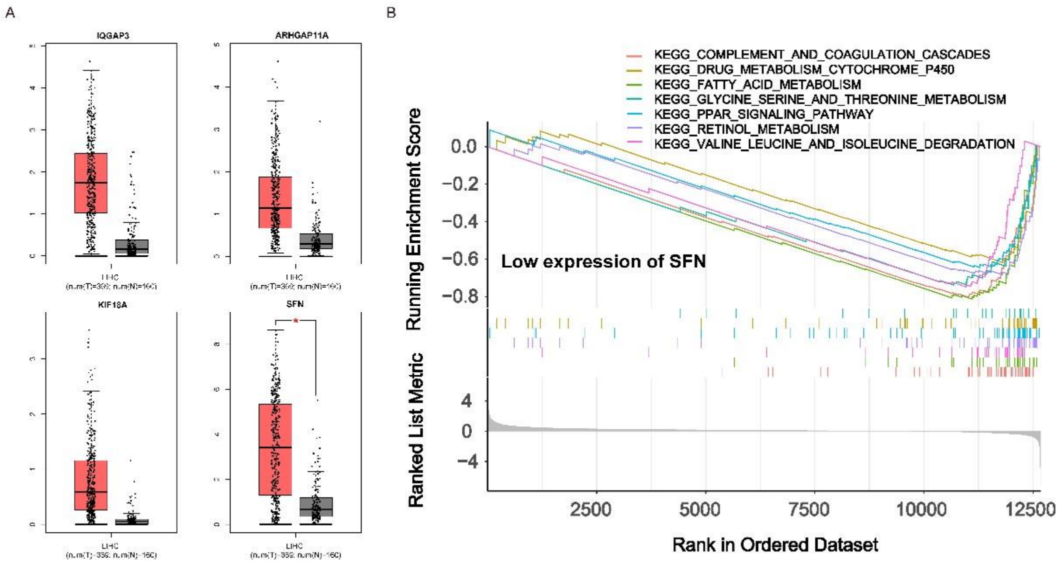

Figure S10. (A) ARHGAP11A, IQGAP3 KIF18A, and SFN expression level between LIHC and normal samples from GEPIA database. LIHC: Liver Hepatocellular Carcinoma. (B) GSEA analysis of patients with low expression SFN.
